# Competing sexual-asexual generic names in *Agaricomycotina* (*Basidiomycota*) with recommendations for use

**DOI:** 10.1186/s43008-021-00061-3

**Published:** 2021-08-11

**Authors:** Joost A. Stalpers, Scott A. Redhead, Tom W. May, Amy Y. Rossman, Jo Anne Crouch, Marc A. Cubeta, Yu-Cheng Dai, Roland Kirschner, Gitta Jutta Langer, Karl-Henrik Larsson, Jonathan Mack, Lorelei L. Norvell, Franz Oberwinkler, Viktor Papp, Peter Roberts, Mario Rajchenberg, Keith A. Seifert, R. Greg Thorn

**Affiliations:** 1 Baarn, The Netherlands; 2 Ottawa Research and Development Centre, Science and Technology Branch, Agriculture and Agri-Food Canada, CEF, Ottawa, Ontario K1A OC6 Canada; 3 Royal Botanic Gardens Victoria, 100 Birdwood Avenue, Melbourne, Victoria 3004 Australia; 4 Department of Botany and Plant Pathology, Oregon State University, Corvallis, OR 97331 USA; 5 USDA-ARS, Mycology & Nematology Genetic Diversity & Biology Laboratory, Beltsville, MD 20705 USA; 6 Department of Entomology and Plant Pathology, North Carolina State University, Raleigh, NC 27606 USA; 7 Beijing Advanced Innovation Center for Tree Breeding by Molecular Design, Beijing Forestry University, Beijing, China; 8 Department of Biomedical Sciences and Engineering, National Central University, Zhongli District, Taoyuan City, 320 Taiwan, Republic of China; 9 Department of Forest Protection, Northwest German Forest Research Institute (NW-FVA), 37079 Goettingen, Lower Saxony Germany; 10 Natural History Museum, University of Oslo, Blindern, 0318 Oslo, Norway; 11 Pacific Northwest Mycology Service, Portland, OR 97229 USA; 12 Lehrstuhl für Spezielle Botanik und Mykologie, Botanisches Institut, Universität, Auf der Morgenstelle 1, 72076 Tübingen, Germany; 13 Department of Botany, Institute of Agronomy, Hungarian University of Agriculture and Life Sciences, Budapest, Hungary; 14 Powys, Wales, UK; 15 Centro Forestal CIEFAP, C.C. 14, 9200 Esquel, Chubut Argentina; 16 National Research Council of Argentina (CONICET), Buenos Aires, Argentina; 17 Department of Biology, Carlton University, Ottawa, Ontario K1S 5B6 Canada; 18 Department of Biology, The University of Western Ontario, London, Ontario N6A 5B7 Canada

**Keywords:** Basidiomycetes, Dual nomenclature, Pleomorphic fungi, Taxonomy, Unit nomenclature, New taxa

## Abstract

With the change to one scientific name for fungal taxa, generic names typified by species with sexual or asexual morph types are being evaluated to determine which names represent the same genus and thus compete for use. In this paper generic names of the *Agaricomycotina* (*Basidiomycota*) were evaluated to determine synonymy based on their type. Forty-seven sets of sexually and asexually typified names were determined to be congeneric and recommendations are made for which generic name to use. In most cases the principle of priority is followed. However, 16 generic names are recommended for use that do not have priority and thus need to be protected: *Aleurocystis* over *Matula; Armillaria* over *Acurtis* and *Rhizomorpha; Asterophora* over *Ugola; Botryobasidium* over *Acladium*, *Allescheriella, Alysidium, Haplotrichum*, *Physospora,* and *Sporocephalium; Coprinellus* over *Ozonium; Coprinopsis* over *Rhacophyllus; Dendrocollybia* over *Sclerostilbum* and *Tilachlidiopsis; Diacanthodes* over *Bornetina; Echinoporia* over *Echinodia; Neolentinus* over *Digitellus; Postia* over *Ptychogaster; Riopa* over *Sporotrichum; Scytinostroma* over *Artocreas, Michenera*, and *Stereofomes; Tulasnella* over *Hormomyces; Typhula* over *Sclerotium;* and *Wolfiporia* over *Gemmularia* and *Pachyma.* Nine species names are proposed for protection: *Botryobasidium aureum, B. conspersum*, *B. croceum*, *B. simile, Pellicularia lembosporum* (syn. *B. lembosporum*), *Phanerochaete chrysosporium*, *Polyporus metamorphosus* (syn. *Riopa metamorphosa*), *Polyporus mylittae* (syn. *Laccocephalum mylittae*), and *Polyporus ptychogaster* (syn*. Postia ptychogaster*). Two families are proposed for protection: *Psathyrellaceae* and *Typhulaceae*. Three new species names and 30 new combinations are established, and one lectotype is designated.

## INTRODUCTION

With the change to one scientific name for fungal taxa in accordance with the *International Code of Nomenclature for algae, fungi, and plants* (McNeill et al. 2012; Turland et al. 2018), there is a need to determine which generic name should be applied when two or more generic names typified by types representing different morphs are congeneric. Formal recommendations about which generic name to use have been made by specialized Subcommissions or Working Groups (WG) of the International Commission for the Taxonomy of Fungi (ICTF) as discussed by May (2017). A number of publications have recommended generic names for use in major groups of *Ascomycota*, including *Sordariomycetes* such as *Diaporthales* (Rossman et al. 2015a), *Hypocreales* (Rossman et al. 2013; Quandt et al. 2014; Kepler et al. 2017), *Magnaporthales* (Zhang et al. 2016), *Microascales* and *Ophiostomatales* (De Beer et al. 2013), *Xylariaceae* (Stadler et al. 2013) and remaining *Sordariomycetes* (Réblová et al. 2016), as well as *Dothideomycetes* (Rossman et al. 2015b), *Eurotiales* (Samson et al. 2014; Visagie et al. 2014), *Leotiomycetes* including *Erysiphales* (Braun 2013; Johnston et al. 2014), *Pezizomycetes* (Healy et al. 2016), yeast fungi (Daniel et al. 2014), and overlooked generic names in the *Ascomycota* (Rossman et al. 2016a). Within the *Basidiomycota* a paper on the *Pucciniomycotina* and *Ustilaginomycotina* has been published (Aime et al. 2018). Where recommended names do not follow the principle of priority, such names need to be confirmed for protection by the Nomenclature Committee for Fungi (NCF) appointed by the International Mycological Congress and ultimately by the General Committee on Nomenclature appointed by the International Botanical Congress. Names approved so far by the NCF have been compiled by May (2017). Once approved, protected names appear in the on-line Appendix to the *International Code of Nomenclature for algae, fungi, and plants* (Wiersema et al. 2020).

Generic names of *Agaricomycotina* representing sexually and asexually typified genera that compete for use have been evaluated by the *Homobasidiomycetes* WG of the ICTF**. The original members of this group were assembled prior to compilation of the list of names needing assessment and once the list was prepared, additional mycologists who have an interest in the nomenclature of this group were involved, leading to the recommendations made herein about which generic name to use. The comprehensive list of sexual-asexual generic names by Wijayawardene et al. (2012) was used as the initial starting point for determining which generic names in the *Agaricomycotina* compete for use. Citations for generic names and their types are based on *Index Fungorum* (Kirk 2020). Each set of generic names was evaluated using the current literature about the phylogenetic placement of their types to determine if the names are congeneric. A recommendation for use is presented based on consideration of such factors as priority, number of species, required name changes, and frequency of citations in the literature based primarily on Google Scholar searches (GSS). Because there are relatively few competing generic names, they are listed in alphabetical order rather than by fungal order^1^.

Forty-seven sets of generic names were identified and evaluated. Details about each set of generic names and the basis for each decision are presented below with an **S** indicating a sexually typified name, an **A** indicating an asexually typified name, and **A/S** indicating either ambiguity or both. The citation for each generic name, their types, accepted name of type, and action required are listed in Table 1. Many generic names recommended for use have priority by date with relatively few or no name changes required. However, 16 generic names recommended for use do not have priority and are thus recommended for protection: *Aleurocystis* over *Matula; Armillaria* over *Acurtis* and *Rhizomorpha; Asterophora* over *Ugola; Botryobasidium* over *Acladium*, *Allescheriella, Alysidium, Haplotrichum*, *Physospora,* and *Sporocephalium; Coprinellus* over *Ozonium; Coprinopsis* over *Rhacophyllus; Dendrocollybia* over *Sclerostilbum* and *Tilachlidiopsis; Diacanthodes* over *Bornetina; Echinoporia* over *Echinodia; Neolentinus* over *Digitellus; Postia* over *Ptychogaster; Riopa* over *Sporotrichum; Scytinostroma* over *Artocreas, Michenera,* and *Stereofomes; Tulasnella* over *Hormomyces; Typhula* over *Sclerotium;* and *Wolfiporia* over *Gemmularia* and *Pachyma.* In addition, nine specific names are proposed for protection: *Botryobasidium aureum, B. conspersum*, *B. croceum*, *B. simile, Pellicularia lembosporum* (syn. *B. lembosporum*), *Phanerochaete chrysosporium*, *Polyporus metamorphosus* (*Riopa metamorphosa*), *P. mylittae* (*Laccocephalum mylittae*) and *P*. *ptychogaster (Postia ptychogaster)* as listed in Table 2. Two family names are proposed for protection: *Psathyrellaceae* over *Zerovaemycetaceae* and *Typhulaceae* over *Sclerotiaceae* as listed in Table 3. Three new species names and 30 new combinations are established and one lectotype is designated.

**Table 1:** Recommended names of genera of *Agaricomycotina* among those that compete for use. The recommended accepted name is in bold; see text for the rationale for these decisions. For each name this list provides the author, its date and place of publication, type, its basionym, their dates of publication and the currently accepted name, if different. The action required is indicated in the last column, specifically approval by the Nomenclature Committee for Fungi (NCF) for those generic names that do not have priority and thus need protection

Recommended generic name	Synonymous alternate morph generic name(s)	Action required
***Aegerita*** Pers. in Neues Mag. Bot. **1**: 120. 1794, nom. sanct., Fr., Syst. Mycol. **1**: xli. 1821. Typus: *A. candida* Pers. 1794, nom. sanct., Fr.	*Crocysporium* Corda, Icon. Fung. **1**: 5. 1837. Typus: *Crocysporium aegerita* Corda 1837, now regarded as *Aegerita candida* Pers. 1794, nom. sanct., Fr. *Bulbillomyces* Jülich in Persoonia **8**: 69. 1974. Typus: *B. farinosus* (Bres.) Jülich 1974, basionym *Kneiffia farinosa* Bres. 1903, now regarded as *Aegerita candida* Pers. 1794, nom. sanct., Fr.	None.
***Aleurocystis*** Lloyd ex G. Cunn. in Trans. Roy. Soc. New Zealand **84**: 234. 1956. Typus: *A. hakgallae* (Berk. & Broome) G. Cunn. 1956, basionym: *Corticium hakgallae* Berk. & Broome 1875.	*Matula* Massee in J. Roy. Microscop. Soc. London, ser. 2 **8**: 176. 1888. Typus: *M. poroniiforme* (Berk. & Broome) Massee 1888, basionym: *Artocreas poroniiforme* Berk. & Broome1875, now regarded as *Aleurocystis hakgallae* (Berk. & Broome) G. Cunn. 1956.	Protection needed by NCF.
***Armillaria*** (Fr.) Staude, Schwämme Mitteldeutschl*.* **28**: 130. 1857, basionym: *Agaricus* "trib." [unranked] *Armillaria* Fr. 1821, nom. sanct. Typus: *A. mellea* (Vahl) P. Kumm. 1871, basionym: *Agaricus melleus* Vahl 1790, nom. sanct., Fr.	*Rhizomorpha* Roth in Ann. Bot. (Usteri) **1**: 7. 1791, nom. sanct., Fr. Typus: *R. fragilis* Roth 1791, now regarded as *Armillaria mellea* (Vahl) P. Kumm. 1871. *Acurtis* Fr., Summa Veg. Scand., Sectio Post. (Stockholm) 337. 1849, now regarded as *Armillaria mellea* (Vahl) P. Kumm. 1871.	Protection needed by NCF.
***Arthrosporella*** Singer in Fl. Neotrop. Monogr. **3**: 17. 1970. Typus: *A. ditopa* (Singer) Singer 1970, basionym: *Armillariella ditopa* Singer 1951.	*Nothoclavulina* Singer in Fl. Neotrop., Monogr. **3**: 18. 1970. Typus: *N. ditopa* Singer 1970, now regarded as *Arthrosporella ditopa* (Singer) Singer 1970.	None.
***Asterophora*** Ditmar in J. Bot. (Schrader) **3**: 56. 1808, nom. sanct., Fr., Syst. Mycol. **3**: 205. 1829. Typus: *A. lycoperdoides* (Bull.) Ditmar 1809, nom. cons., basionym: *Agaricus lycoperdoides* Bull. 1784., nom. cons.	*Ugola* Adans., Fam. Pl. **2**: 5. 1763. Typus: *Asterophora physaroides* Fr. 1817, nom. sanct., Fr., synonym: *Ugola physaroides* (Fr.) Redhead & Seifert 2001, now regarded as *Asterophora lycoperdoides* (Bull.) Ditmar 1809. *Nyctalis* Fr., Syst. Orb. Veg. **1**: 78. 1825. Typus: *N. parasitica* (Bull.) Fr. 1838, basionym: *Agaricus parasiticus* Bull. 1791, nom. sanct., Fr., now *Asterophora parasitica* (Bull.) Singer 1951.	Protection needed by NCF.
***Athelia*** Pers., Traité Champ. Comest. 57. 1818. Typus: *A. epiphylla* Pers. 1818 (*Thelephora epiphylla* (Pers.) Fr., nom sanct., Fr.).	*Fibularhizoctonia* G.C. Adams & Kropp in Mycologia **88**: 464. 1996. Typus: *F. carotae* (Rader) G.C. Adams & Kropp 1996, basionym *Rhizoctonia carotae* Rader 1948, now regarded as *Athelia arachnoidea* (Burt) Jülich 1972.	None.
***Bjerkandera*** P. Karst. in, *Meddelanden af Societas pro Fauna et Flora Fennica* **5**: 38. 1879. Typus: *Bjerkandera adusta* (Willd.) P. Karst. 1879, basionym: *Boletus adustus* Willd. 1878, nom. sanct., Fr.	*Geotrichopsis* Tzean & Estey in Mycological Research **95**: 1351. 1991. Typus: *G. mycoparasitica* Tzean & Estey 1991.	None
***Botryobasidium*** Donk in Meded. Ned. Mycol. Ver. **18–20**: 116. 1931. Typus: *B*. *subcoronatum* (Höhn. & Litsch.) Donk 1931, basionym: *Corticium subcoronatum* Höhn. & Litsch. 1907.	*Acladium* Link in Ges. Naturf. Freunde Berlin Mag. **3**: 11. 1809. Typus: *A. conspersum* Link 1809, now regarded as *Botryobasidium conspersum* J. Erikss. 1958. *Alysidium* Kunze in Kunze & Schmidt, Mykol. Hefte **1**: 11. 1817. Typus: *A. fulvum* Kunze & J.C. Schmidt 1817, nom. sanct., Fr., now regarded as *Botryobasidium aureum* Parmasto 1965. *Haplotrichum* Link in Willd., Sp. Pl. **6**(1): 52. 1824. Typus: *H. capitatum* Link 1824, now regarded as *Botryobasidium capitatum* (Link) Rossman & W.C. Allen 2016. *Sporocephalium* Chevall., Fl. Gén. Env. Paris **1**: 59. 1826. Typus: *S. capitatum* (Link) Chevall. 1826, basionym: *Haplotrichum capitatum* Link. 1824, now regarded as *Botryobasidium capitatum* (Link) Rossman & W.C. Allen 2016. *Physospora* Fr., Fl. Scan. 360. 1837. Typus: *Sporotrichum rubiginosum* Fr. 1832, now regarded as. *Botryobasidium rubiginosum* (Fr.) W.C. Allen & Rossman 2016. *Allescheriella* Henn. in Hedwigia **36**: 244. 1897. Typus: *A. uredinioides* Henn. 1897, now regarded as *Botryobasidium croceum* Lentz 1967. *Neoacladium* P.N. Singh & S.K. Singh in Fungal Diversity **96**: 189. 2019. Typus: *N. indicus* P.N. Singh & S.K. Singh 2019, now regarded as *Botryobasidium indicum* (P.N. Singh & S. K Singh) R. Kirschner & G. Langer 2021.	Protection needed by NCF.
***Bullera*** Derx in Ann. Mycol*.* **28**: 11. 1930. Typus: *B. alba* (W.F. Hanna) Derx 1930, basionym: *Sporobolomyces albus* W.F. Hanna 1929.	*Bulleromyces* Boekhout & Á. Fonseca, in Antonie van Leeuwenhoek **59**: 91. 1991. Typus: *B. albus* Boekhout & Á. Fonseca 1991, now regarded as *Bullera alba* (W.F. Hanna) Derx 1930.	None.
***Chaetospermum*** Sacc., Syll. Fung. **10**: 706. 1892. Typus: *C. chaetosporum* (Pat.) A.L. Sm. & Ramsb. 1913, basionym: *Tubercularia chaetospora* Pat. 1888.	*Efibulobasidium* K. Wells in Mycologia **67**: 148. 1975. Typus: *E. albescens* (Sacc. & Malbr.) K. Wells 1975, basionym: *Epidochium albescens* Sacc. & Malbr. 1881, now regarded as *Chaetospermum gossypinum* (G.F. Atk.) Nag Raj 1993.	None.
***Coprinellus*** P. Karst. in Bidrag Kännedom Finlands Natur Folk **32**: 542. 1879. Typus: *C. deliquescens* (Bull.) P. Karst. 1879, basionym: *Agaricus deliquescens* Bull. 1786, nom. sanct., Fr.	*Ozonium* Link in Ges. Naturf. Freunde Berlin Mag. **3**: 21. 1809. Typus: *O. auricomum* Link 1809, now regarded as *Coprinellus domesticus* (Bolton) Vilgalys et al. 2001.	Protection needed by NCF.
***Coprinopsis*** P. Karst. in Acta Soc. Fauna Fl. Fenn. **2**: 27. 1881, nom. cons. Typus: *C. friesii* (Quél.) P. Karst. 1881, basionym: *Coprinus friesii* Quél. 1872.	*Rhacophyllus* Berk. & Broome in J. Linn. Soc., Bot. **11**: 559. 1871. Typus: *R. lilacinus* Berk. & Broome 1871, now regarded as *Coprinopsis lilacina* (Berk. & Broome) Redhead 2021. *Zerovaemyces* Gorovij, Dokl. Akad. Nauk Ukrainskoĭ SSR, Ser. B **39**(8): 745. 1977. Typus: *Zerovaemyces copriniformis* Gorovij, 1977, now regarded as *Coprinopsis lilacina* (Berk. & Broome) Redhead 2021. *Hormographiella* Guarro & Gené in Mycotaxon **45**: 179. 1992. Typus: *H. aspergillata* Guarro, Gené & De Vroey 1992, now regarded as *Coprinopsis cinerea* (Schaeff.) Redhead, et al. 2001.	Protection needed by NCF.
***Cryptococcus*** Vuill. in Rev. Gén. Sci. Pures Appl. **12**: 741. 1901, nom. cons. Typus: *C. neoformans* (San Felice) Vuill. 1901, basionym: *Saccharomyces neoformans* San Felice 1895, nom. cons.	*Filobasidiella* Kwon-Chung in Mycologia **67**: 1198. 1976. "1975". Typus: *F. neoformans* Kwon-Chung 1976, now regarded as *Cryptococcus neoformans* (San Felice) Vuill. 1901.	None.
***Dacrymyces*** Nees, Syst. Pilze 89. 1816 [1816–17], nom. sanct., Fr., Syst. Mycol. **2**: 228. 1822. Typus: *D. stillatus* Nees 1816, nom. sanct., Fr.	*Ditiola* Fr., Syst. Mycol*.* **2**: 39. 1822, nom. sanct., Fr., non *Ditiola* P. Browne 1756 = *Schizophyllum* Fr. 1815. Typus: *D. radicata* (Alb. & Schwein.) Fr. 1822, nom. sanct., basionym: *Helotium radicatum* Alb. & Schwein. 1805, now regarded as *Dacrymyces radicatus* (Alb. & Schwein.) Donk 1931. *Dacryoscyphus* R. Kirschner & Zhu L. Yang in Antonie van Leeuwenhoek **87**: 331. 2005. Typus: *D. chrysochilus* R. Kirschner & Zhu L. Yang 2005. *Pionnotes* Fr., Summa Veg. Scand. 481. 1849. Typus: *P. capitata* (Schwein.) Fr. 1849, basionym: *Fusarium capitatum* Schwein. 1832, now regarded as *Dacrymyces chrysospermus* Berk. & M.A. Curtis 1873.	None.
***Deconica*** (W.G. Sm.) P. Karst. in Bidrag Kännedom Finlands Natur Folk **32**: 515. 1879, basionym: *Agaricus* subgen. *Deconica* W.G. Sm. 1870. Typus: *D. bullacea* (Bull.) Sacc. 1887, basionym: *Agaricus bullaceus* Bull. 1793, nom. sanct., Fr.	*Pseudohelicomyces* Garnica & E. Valenz. in Mycol. Res. **104**: 739. 2000. Typus: *P. albus* Garnica & E. Valenz. 2000, now regarded as *Deconica merdaria* (Fr.) Noordel. 2009.	None.
***Dendrocollybia*** R.H. Petersen & Redhead in Mycol. Res. **105**: 169. 2001. Typus: *D. racemosa* (Pers.) R.H. Petersen & Redhead 2001, basionym: *Agaricus racemosus* Pers. 1797, nom. sanct., Fr.	*Tilachlidiopsis* Keissl. in Ann. Naturhist. Mus. Wien **37**: 215. 1924. Typus: *T. racemosa* Keissl. 1924, now regarded as *Dendrocollybia racemosa* (Pers.) R.H. Petersen & Redhead 2001. *Sclerostilbum* Povah in Mycologia **24**: 242. 1932. Typus: *S. septentrionale* Povah 1932, now regarded as *Dendrocollybia racemosa* (Pers.) R.H. Petersen & Redhead 2001.	Protection needed by NCF.
***Diacanthodes*** Singer in Lloydia **8**: 141. 1945. Typus: *D. philippinensis* (Pat.) Singer 1945, basionym: *Daedalea philippinensis* Pat. 1915, now regarded as *Diacanthodes novoguineensis* (Henn.) O. Fidalgo 1962.	*Bornetina* L. Mangin & Viala in Compt. Rend. Hebd. Séances Acad. Sci. **136**: 398. 1903. Typus: *B. corium* L. Mangin & Viala 1903, now regarded as *Diacanthodes novoguineensis* (Henn.) O. Fidalgo 1962.	Protection needed by NCF.
***Ditangium*** P. Karst., Fungi Fenniae Exsiccati **7**: 656. 1867. Typus: *D. insigne* P. Karst. 1870.	*Craterocolla* Bref. in Unters. Gesammtgeb. Mykol. **7**: 98. 1888. Typus: *C. cerasi* (Schumach.) Sacc. 1888, basionym *Tremella cerasi* Schumach. 1803, now regarded as *Ditangium cerasi* (Schumach.) Constantin & J.L. Dufour 1891. *Poroidea* Göttinger ex G. Winter, Rabenh. Krypt.-Fl., ed. 2 **1**, 2: 275. 1885. Typus: *P. pithyophila* Göttinger ex G. Winter 1885, now regarded as *Ditangium cerasi* (Schumach.) Constantin & J.L. Dufour 1891.	None.
***Echinoporia*** Ryvarden in Ryvarden & Johansen, Prelim. Polyp. Fl. East Africa 325. 1980. Typus: *E. hydnophora* (Berk. & Broome) Ryvarden 1980.	*Echinodia* Pat. in Bull. Soc. Mycol. France **34**: 199. 1918. Typus: *E. theobromae* Pat. 1918, now regarded as *Echinoporia hydnophora* (Berk. & Broome) Ryvarden 1980.	Protection needed by NCF.
***Femsjonia*** Fr., Summa Veg. Scand*.* 341. 1849. Typus: *F. luteoalba* Fr. 1849, now regarded as *Femsjonia peziziformis* (Lév.) P. Karst. 1876, basionym *Exidia peziziformis* Lév. 1848.	*Cerinosterus* R.T. Moore in Stud. Mycol. **30**: 216. 1987. Typus: *C. luteoalbus* (de Hoog) R.T. Moore 1987, basionym: *Sporothrix luteoalba* de Hoog 1974, now regarded as a synonym of *Femsjonia peziziformis* (Lév.) P. Karst. 1876.	None.
***Fistulina*** Bull., Hist. Champ. France 313. 1791, nom. sanct., Fr., Syst. Mycol. **1**: 396. 1821. Typus: *F. buglossoides* Bull. 1791, now regarded as *Fistulina hepatica* (Schaeff.) With. 1801, nom. sanct., Fr.	*Confistulina* Stalpers in Canad. J. Bot. **61**: 1660. 1983. Typus: *C. hepatica* (Sacc.) Stalpers 1983, basionym: *Ceriomyces hepaticus* Sacc. 1888, now regarded as *Fistulina hepatica* (Schaeff.) With. 1801.	None.
***Heteroacanthella*** Oberw. in Trans. Mycol. Soc. Japan **31**: 208. 1990. Typus: *H. variabilis* Oberw. & Langer 1990.	*Acanthellorhiza* P. Roberts, Rhizoctonia-Forming Fungi 130. 1998. Typus: *A. globulifera* P. Roberts 1999, now regarded as *Heteroacanthella acanthophysa* (Burds.) Oberw. 1990.	None.
***Heterobasidion*** Bref. in Unters. Gesammtgeb. Mykol. **8**: 154. 1888. Typus: *H. annosum* (Fr.) Bref. 1888, basionym: *Polyporus annosus* Fr. 1821, nom. sanct.	*Spiniger* Stalpers in Proc. Kon. Ned. Akad. Wetensch. C **77**: 402. 1974. Typus: *S. meineckellus* (A.J. Olson) Stalpers 1974, basionym: *Cunninghamella meineckella* A.J. Olson 1941, now regarded as *Heterobasidion annosum* (Fr.) Bref. 1888.	None.
***Hohenbuehelia*** Schulzer in Verh. K. K. Zool.-bot. Ges. Wien **16**: 45. 1866. Typus: *H. petaloides* (Bull.) Schulzer 1866, basionym: *Agaricus petaloides* Bull. 1785, nom. sanct., Fr.	*Nematoctonus* Drechsler in Phytopathology **31**: 779. 1941. Typus: *N. tylosporus* Drechsler 1941, now regarded as *Hohenbuehelia tylospora* (Drechsler) Thorn 2013.	None.
***Leucocoprinus*** Pat. in J. Bot. (Morot) **2**: 16. 1888. Typus: *L. cepistipes* Pat. 1889	*Attamyces* Kreisel in Z. Allg. Mikrobiol. Morphol. Physiol. Ökol. Mikroorgan*.* **12**: 648. 1972. Typus: *A. bromatificus* Kreisel 1972, now regarded as *Leucocoprinus gongylophorus* (Möller) R. Heim 1957.	None.
***Marchandiomyces*** Diederich & D. Hawksw. in Mycotaxon **37**: 311. 1990. Typus: *M. corallinus* (Roberge) Diederich & D. Hawksw. 1990, basionym: *Illosporium corallinum* Roberge 1847.	*Marchandiopsis* Ghobad-Nejhad & Hallenb. in Taxon **59**: 1530. 2010. Typus: *M. quercina* (J. Erikss. & Ryvarden) Ghobad-Nejhad & Hallenb., basionym *Laeticorticium quercinum* J. Erikss. & Ryvarden 1976., now *Marchandiomyces quercinus* (J. Erikss. & Ryvarden) Diederich & D. Hawksw. 2015.	None
***Mycena*** (Pers.) Roussel, Fl. Calvados, ed. 2 64 (‘46’). 1806, basionym: *Agaricus* sect. *Mycena* Pers. 1797. Typus: *M. galericulata* (Scop.) Gray 1821, basionym: *Agaricus galericulatus* Scop. 1772, nom. sanct., Fr.	*Decapitatus* Redhead & Seifert in Taxon **49**: 795. 2000. Typus: *D. flavidus* (Cooke) Redhead & Seifert 2000, basionym: *Stilbum flavidum* Cooke 1880, now *Mycena citricolor* (Berk. & M.A. Curtis) Sacc. 1887.	None.
***Myxarium*** Wallr., Fl. Crypt. Germ. **2**: 260. 1833. Typus: *M. nucleatum* Wallr. 1833.	*Hyaloria* Möller in Bot. Mitt. Tropen **8**: 137. 1895. Typus: *H. pilacre* Möller 1895, now regarded as *Myxarium pilacre* (Möller) R. Kirschner 2018. *Helicomyxa* R. Kirschner & Chee J. Chen in Stud. Mycol. **50**: 338. 2004. Typus: *H. everhartioides* R. Kirschner & Chee J. Chen 2004, now regarded as *Myxarium everhartioides* (R. Kirschner & Chee J. Chen) R. Kirschner 2018.	None.
***Necator*** Massee, Bull. Misc. Inf., Kew **1898**: 119. 1898. Typus: *N. decretus* Massee, 1898.	*Upasia* Harsojo-Tjokrosoedarmo & Rifai. Ilmu Pertanian (Agric. Sci.) **5**(1): 566. 1992. Typus: *U. salmonicolor* (Berk. & Broome) Harsojo-Tjokrosoedarm, now regarded as *Necator salmonicolor* (Massee) K.H. Larss. et al. 2021.	None.
***Neolentinus*** Redhead & Ginns, Trans. Mycol. Soc. Japan **26**(3): 357. 1985. Typus: *N. kauffmanii* (A.H. Sm.) Redhead & Ginns 1985, basionym: *Lentinus kauffmanii* A.H. Sm. 1946.	*Digitellus* Paulet, Traité champ. (Paris) **2**: 420, [485] and in Index. 1793. Typus: *Digitellus humanus* Paulet 1793, now regarded as *Neolentinus lepideus* (Fr.) Redhead & Ginns 1985.	Protection needed by NCF.
***Oliveonia*** Donk in Fungus **28**: 20. 1958. Typus: *O. fibrillosa* (Burt) Donk 1958, basionym: *Sebacina fibrillosa* Burt 1926.	*Oliveorhiza* P. Roberts in Folia Cryptog. Estonica **33**: 128. 1998. Typus: *O. anapauxilla* P. Roberts 1998, now regarded as *Oliveonia pauxilla* (H.S. Jacks.) Donk 1958.	None.
***Pleurotus*** (Fr.) P. Kumm., Führ. Pilzk. (Zerbst) 104. 1871, basionym: *Agaricus* "trib." [unranked] *Pleurotus* Fr. 1821, nom. sanct.. Typus: *P. ostreatus* (Jacq.) P. Kumm. 1871, basionym: *Agaricus ostreatus* Jacq. 1774, nom. sanct., Fr.	*Antromycopsis* Pat. & Trab. in Bull. Soc. Mycol. France **13**: 215. 1897. Typus: *A. broussonetiae* Pat. & Trab., 1897, now regarded as *Pleurotus cystidiosus* O.K. Mill. 1969.	None.
***Polyporus*** P. micheli ex Adans., Fam. Pl. **2**: 10. 1763, nom. sanct. Fr., Syst. Mycol. 1: 341. 1821. Typus: *P. tuberaster* (Jacq. ex Pers.) Fr. 1815, nom. sanct. Fr., basionym: *Boletus tuberaster* Jacq. ex Pers. 1801.	*Mycelithe* Gasp. in Atti Accad. Pontan*.* **2**: 221. 1842. Typus: *M. fungifera* Gasp. 1842, now regarded as *Polyporus tuberaster* (Jacq. ex Pers.) Fr. 1815.	None.
***Postia*** Fr., Hymenomyc. Eur. 586. Oct 1874. Typus: *Polyporus lacteus* Fr. 1821, nom. sanct., now *Postia lactea* (Fr.) P. Karst. 1881.	*Ptychogaster* Corda, Icon. Fung*.* **2:** 23. 1838. Typus: *P. albus* Corda 1838, now regarded as *Postia ptychogaster* (F. Ludw.) Vesterh. 1996.	Protection needed by NCF.
***Rhizoctonia*** DC. in Lamarck & de Candolle, Fl. Franç., ed. 3 **5**: 110. 1815. Typus: *R. solani* J.G. Kühn 1858.	*Thanatephorus* Donk in Reinwardtia **3**: 376. 1956. Typus: *T. cucumeris* (A.B. Frank) Donk 1956, basionym: *Hypochnus cucumeris* A.B. Frank 1883, now regarded as *Rhizoctonia solani* J.G. Kühn 1858.	None.
***Riopa*** D.A. Reid in Revue Mycol., Paris **33**: 244. 1969. Typus: *Riopa davidii* D.A. Reid 1969, now regarded as *Riopa metamorphosa* (Fuckel) Miettinen & Spirin 2016, basionym: *Polyporus metamorphosus* Fuckel in Jahrb. Nassauischen Vereins Naturk. **27–28**: 87. 1874.	*Sporotrichum* Link in Ges. Naturf. Freunde Berlin Mag. **3:** 13. 1809. Typus: *S. aureum* Link 1809 nom. illeg. non (Pers.) Fr. 1832 [= *Trichoderma aureum* Pers. 1796 = *Botryobasidium aureum* Parmasto 1965], now regarded as *Riopa metamorphosa* (Fuckel) Miettinen & Spirin 2016.	Protection needed by NCF.
***Scytinostroma*** Donk in Fungus **26**: 19. 1956. Typus: *S. portentosum* (Berk. & M.A. Curtis) Donk 1956, basionym: *Corticium portentosum* Berk. & M.A. Curtis 1873.	*Michenera* Berk. & M.A. Curtis in J. Linn. Soc., Bot. **10**: 333. 1868. "1869". Typus: *M. artocreas* Berk. & M.A. Curtis 1868, now regarded as *Scytinostroma artocreas* (Berk. & M.A. Curtis) K.-H. Larss. 2018. *Artocreas* Berk. & Broome, J. Linn. Soc., Bot. **14**(74): 73. 1873 "1875". Typus: *Artocreas micheneri* Berk. & M.A. Curtis. 1875, now regarded as as *Scytinostroma artocreas* (Berk. & M.A. Curtis) K.-H. Larss. 2018. *Stereofomes* Rick in Egatea **13**: 435. 1928. Typus: *S. nodulosum* Rick 1928, now regarded as *Scytinostroma nodulosum* (Rick) K-H Larss. 2018. *Licrostroma* P.A. Lemke in Canad. J. Bot. **42**: 762. 1964. Typus: *L. subgiganteum* (Berk.) P.A. Lemke 1964, basionym: *Corticium subgiganteum* Berk. 1873, now regarded as *Scytinostroma artocreas* (Berk. & M.A. Curtis) K.-H. Larss. 2018.	Protection needed by NCF.
***Sistotrema*** Fr., Syst. Mycol. **1**: 426. 1821, nom. sanct. Typus: *S. confluens* Pers. 1794, nom. sanct., Fr.	*Ingoldiella* D.E. Shaw in Trans. Brit. Mycol. Soc. **59**: 258. 1971. Type: *I. hamata* D.E. Shaw 1971, now regarded as *Sistotrema hamatum* Nawawi & J. Webster 1982.	None.
***Sterigmatosporidium*** G. Kraep. & U. Schulze in Antonie van Leeuwenhoek **48**: 479. 1981 "1982". Typus: *S. polymorphum* G. Kraep. & U. Schulze 1983.	*Cuniculitrema* J.P. Samp. & R. Kirschner in Antonie van Leeuwenhoek **80**: 155. 2001. Typus: *C. polymorpha* R. Kirschner & J.P. Samp. 2001.	None.
***Subulicystidium*** Parmasto, Consp. System. Corticiaceae (Tartu) 120. 1968. Typus: *S. longisporum* (Pat.) Parmasto 1968, basionym: *Hypochnus longisporus* Pat. 1894.	*Aegeritina* Jülich in Int. J. Mycol. Lichenol. **1**: 282. 1984. Typus: *A. tortuosa* (Bourdot & Galzin) Jülich 1984, basionym: *Aegerita tortuosa* Bourdot & Galzin 1928, now regarded as *Subulicystidium longisporum* (Pat.) Parmasto 1968.	None.
***Tomophagus*** Murrill in Torreya **5**: 197. 1901. Typus: *T. colossus* (Fr.) Murrill 1901, basionym *Polyporus colossus* Fr. 1851.	*Thermophymatospora* Udagawa et al. in Mycotaxon **27**: 100. 1986. Typus: *T. fibuligera* Udagawa, et al. 1986, now regarded as *Tomophagus colossus* (Fr.) Murrill 1905.	None.
***Trechispora*** P. Karst. in Hedwigia **29**: 147. 1890. Typus: *T. onusta* P. Karst. 1890, now regarded as *Trechispora hymenocystis* (Berk. & Broome) K.H. Larss. 1994.	*Osteomorpha* G. Arnaud ex Watling & W.B. Kendr. in Naturalist (Hull) ser. 3 **104**: 1. 1978. Typus: *O. fragilis* G. Arnaud ex Watling & W.B. Kendr. 1979, now regarded as *Trechispora stevensonii* (Berk. & Broome) K.H. Larss. 1995.	None.
***Trimorphomyces*** Bandoni & Oberw. in Syst. Appl. Microbiol. **4**: 106. 1983. Typus: *T. papilionaceus* Bandoni & Oberw. 1983.	*Anastomyces* W.P. Wu et al. in Mycol. Res. **101**: 1318. 1997. Typus: *A. microsporus* W.P. Wu, et al. 1997, now regarded as *Trimorphomyces papilionaceus* Bandoni & Oberw. 1983.	None.
***Tulasnella*** J. Schröt. in Cohn, Krypt.-Fl. Schlesien **3**(1): 397. June 1888. Typus: *T. lilacina* J. Schröt. 1888, now regarded as *Tulasnella violea* (Quél.) Bourd. & Galz. 1909, basionym *Hypochnus violeus* Quél. 1883.	*Hormomyces* Bonord., Handb. Mykol. 150. 1851. Typus: *H. aurantiacus* Bonord. 1851, now regarded as *Tulasnella aurantiaca* (Bonord.) J. Mack & Seifert 2021. *Prototremella* Pat. in J. Bot. (Morot) **2**: 269. 1888. Typus: *P. tulasnei* Pat. August 1888, now regarded as *Tulasnella tulasnei* (Pat.) Juel 1897. *Hormisciopsis* Sumst. in Mycologia **6**: 32. 1914. Typus: *H. gelatinosa* Sumst. 1914, now regarded as *Tulasnella aurantiaca* (Bonord.) J. Mack & Seifert 2021. *Epulorhiza* R.T. Moore in Mycotaxon **29**: 94. 1987. Typus: *E. repens* (G.E. Bernard) R.T. Moore 1987, basionym: *Rhizoctonia repens* G.E. Bernard 1909, now regarded as *Tulasnella deliquescens* (Juel) Juel 1914.	Protection needed by NCF.
***Typhula*** (Pers.) Fr., Observ. Mycol*.* **2**: 296. 1818, basionym: *Clavaria* y *Typhula* Pers. 1801. Typus: *T. phacorrhiza* (Reichard) Fr. 1818, nom. sanct., Fr., basionym: *Clavaria phacorrhiza* Reichard 1780.	*Sclerotium* Tode, Fung. Mecklenb. Sel. **1**: 2. 1790, nom. sanct., Fr. Typus: *S. complanatum* Tode 1790, now regarded as *Typhula phacorrhiza* (Reichard) Fr. 1818.	Protection needed by NCF.
***Waitea*** Warcup & P.H.B. Talbot in Trans. Br. Mycol. Soc. **45**: 503. 1962. Typus: *W. circinata* Warcup & P.H.B. Talbot 1962.	*Chrysorhiza* T.F. Andersen & Stalpers in Sneh et al., Rhizoctonia Species, Taxonomy, Molecular Biology, Ecology, Pathology and Disease Control (Dordrecht): 58. 1996. Typus: *Rhizoctonia zeae* Voorhees 1934, now regarded as *Waitea zeae* (Voorhees) J.A. Crouch & Cubeta 2021.	None.
***Wolfiporia*** Ryvarden & Gilb. in Mycotaxon **19**: 141. 1984. Typus: *W. cocos* (F.A. Wolf) Ryvarden & Gilb. 1984, basionym: *Poria cocos* F.A. Wolf 1922, nom. cons.	*Gemmularia* Raf. in J. Phys. Chim. Hist. Nat. Arts **89**: 106. 1819, nom. sanct., Fr., 1823. Typus: *G. rugosa* Raf. 1819, now regarded as *Wolfiporia cocos* (F.A. Wolf) Ryvarden & Gilb. 1984. *Pachyma* Fr., Syst. Mycol. **2**: 242. 1822, nom. sanct. Typus: *P. cocos* Fr. 1822, basionym: *Sclerotium cocos* Schwein. 1822, now regarded as *Wolfiporia cocos* (F.A. Wolf) Ryvarden & Gilb. 1984. *Tucahus* Raf., Medical flora, or, Manual of the medical botany of the United States of North America. **2**: 270. 1830. Typus: *T. rugosus* (Raf.) Raf., now regarded as *Wolfiporia cocos* (F.A. Wolf) Ryvarden & Gilb. 1984.	Protection needed by NCF.

**Table 2:** Species names proposed for protection with the names proposed for rejection. See text for the rationale about the proposed protected names. For each name this list provides the currently accepted name, if different, the name to be protected and rejected, author, place and date of publication, and type. The names proposed for protection will be evaluated and recommended for approval by the Nomenclature Committee for Fungi

Names proposed for protection	Rejected names
***Botryobasidium conspersum*** J. Erikss. in Symb. Bot. Upsal. **16**: 133. 1958. Typus: Sweden: *Gästrikland*: Gäyle, Lövudden, on decayed wood of *Betula*, 25 Jun 1951, *J A Nannfeldt 11434a* (UPS)	*Acladium conspersum* Link in Ges. Naturf. Freunde Berlin Mag. **3**: 11. 1809, nom. sanct, Fr., Syst. Mycol. **3**: 419. 1832.
	*Sporotrichum oosporum* Ehrenb., Sylv. Mycol. Berol. 22. 1818.
	*Sporotrichum helvolum* Wallr., Fl. Crypt. Germ. **2**: 280. 1833.
	*Sporotrichum floccosum* Bres. in Hedwigia **35**: 301. 1896.
	*Rhinotrichum olivaceum* Bres., Fung. Trident*.* **2**: 106. 1900.
	*Rhinotrichum bicolor* Sumst. in Mycologia **3**: 50. 1911.
	*Rhinotrichum noblesiae* Sumst. in Mycologia **29**: 250. 1937.
***Botryobasidium croceum*** Lentz in Mycopathol. Mycol. Appl. **32**: 6. 1966 [1967]. Typus: USA: Mississippi. Greenville, near Huntington Point, on dead stump, 1960, *P.L. Lentz 60–394* (BPI 1107361 – holotype).	*Mucor croceus* Mont. in Sagra, Ann. Sci. Nat., Bot. Sér. 2 **17**: 121. 1842.
	*Gymnosporium fulvum* Berk. & M.A. Curtis in Berkeley, J. Linn. Soc., Bot. **10**: 355. 1868 "1869".
	*Allescheriella uredinioides* Henn. in Hedwigia **36**: 244. 1897.
***Laccocephalum mylittae*** (Cooke & Massee) Núñez & Ryvarden. Syn. Fung. (Oslo) **10**: 31. 1995 (*Polyporus mylittae* Cooke & Massee in Cooke, Grevillea **21**: 37. 1892). Typus: [Australia, Victoria, Beechworth, *J.W. Howard*], “Growing on *Mylitta australis*. S. Australia [sic]” (K(M)).	*Mylitta australis* Berk. in Ann. Mag. Nat. Hist. **3**: 326. 1839.
	Typus: [Australia, Tasmania], “Van Diemen’s Land, collection of Sir. W.J. Hooker”.
***Phanerochaete chrysosporium*** Burds. in Mycotaxon **1**: 124. 1974. Typus: U.S.A.: Arizona, Cochise Co., Peloncillo Mts., Guadelupe Canyon, on dead wood of *Platanus wrightii* (Arizona sycamore), 25 Aug. 1971, *Burdsall 6251* (Holotype: CFMR; Isotype: Same data ARIZ – AN 003206).	*Sporotrichum pruinosum* Gilman & E.V. Abbott in Iowa State Coll. J. Sci. **1**: 306. 1927.
***Postia ptychogaster*** (F. Ludw.) Vesterh. in Knudsen & Hansen, Nordic J. Bot. **16**: 213. 1996 (*Polyporus ptychogaster* F. Ludw. in Z. Gesammten Naturwiss (Halle) **3**: 424. 1880). Typus: Taf. XIII in Z. Gesammten Naturwiss (Halle) 1880. Lectotypus designated here: MBT 395397.	*Trichoderma fuliginoides* Pers., Syn. Meth. Fung. **1**: 231. 1801.
	*Ptychogaster albus* Corda, Icon. Fung. **2**: 24. 1838.
***Riopa metamorphosa*** (Fuckel) Miettinen & Spirin in MycoKeys **17**: 27. 2016 (*Polyporus metamorphosus* Fuckel in Jahrb. Nassauischen Vereins Naturk. 27-28: 87. 1874). Typus: Germany: *Oestrich (Nassau)*: Mittelheimer Vorderwald, rotten trunk of *Quercus*, “Herbier Fuckel 1894, Herbier Barbey-Boissier”, no. 2008 (S F43290 – lectotype designated by Miettinen et al. 2016). Czech Republic. *Moravia*: Lanžhot, Ranšpurk virgin forest, rotten trunk of *Quercus robur*, 5 Oct 1988, *Z. Pouzar* (PRM 871894 – epitype designated by Miettinen et al. 2016; H 7008579 – isoepitype).	*Mucor aurantius* Bull., Hist. Champ. France **1**: 103. 1791.
	*Sporotrichum aurantiacum* Fr., Syst. Mycol. **3**: 423. 1832, nom. sanct.

**Table 3:** Family names proposed for protection with the names proposed for rejection. See text for the rationale about the proposed protected names. This list provides the names to be protected or rejected, author, their place and date of publication, and type. The names proposed for protection will be evaluated and recommended for approval by the Nomenclature Committee for Fungi

Names proposed for protection	Rejected names
***Psathyrellaceae*** Vilgalys, Moncalvo & Redhead in Taxon **50**: 226. 2001. Typus: *Psathyrella* (Fr.) Quél.	*Zerovaemycetaceae* Gorovij in Dopov. Akad. Nauk URSR, Ser. B **39**(8): 745. 1977. Typus: *Zerovaemyces* Gorovij
***Typhulaceae*** Jülich in Biblioth. Mycol. **85**: 393. 1982 [1981]. Typus: *Typhula* (Pers.) Fr.	*Sclerotiaceae* Dumort. [as ‘Sclerotaceae’], Comment. Bot. (Tournay) 69. 1822. Typus: *Sclerotium* Tode, nom. sanct., Fr.

## GENERIC AND FAMILY NAMES RECOMMENDED FOR USE IN *AGARICOMYCOTINA*

### Use *Aegerita* Pers. 1794 (A) rather than *Crocysporium* Corda 1837 or *Bulbillomyces* Jülich 1974 (S)

The monotypic genus *Bulbillomyces,* typified by *B. farinosus,* was described as the sexual morph of *A. candida* (Jülich 1974), the type of *Aegerita,* thus these generic names are synonyms. Lyman (1907) had earlier demonstrated the link between the sexual and asexual morphs using cultures and transferred *Aegerita candida* to *Peniophora* as *P. candida* (Pers.) Lyman 1907. Although two species were originally included in the protologue of *Aegerita*, Donk (1962) explains the history and selection of *A. candida* as type by Brongniart (1824, 1825), although Donk’s interpretation of validation dates he used in 1962 must be adjusted by application of the current Shenzhen Code (Turland et al. 2018). Forty names have been described in *Aegerita*, although some of these have been found to belong outside of *Aegerita* in both *Ascomycota* and *Basidiomycota* (Kirk 2020). Within *Aegerita* only *A. candida* (as *B. farinosus*) has been sequenced. Recently Justo et al. (2017) showed that *A. candida* (as *B. farinosus*) was sister to the type of *Hypochnicium*, *H. bombycinum*. The name *Aegerita candida* is used about equally with *B. farinosus* (GSS *A. candida* = 131, *B. farinosus* = 105). *Aegerita candida* is the name used by ecologists who have examined the extracellular enzymatic activity of this aero-aquatic fungus (Abdullah and Taj-Aldeen 1989). An obscure generic name, *Crocysporium* (GSS 32), is typified by *C. aegerita*, a name that is considered a synonym of *Aegerita candida* (Donk 1962), thus *Crocysporium* is a later synonym of *Aegerita*. Five other species names have been described in *Crocysporium* of which all but two are placed elsewhere and none have been widely used. Given its priority, the greater number of names, and use in ecological literature, we recommend the use of *Aegerita*.

### Protect *Aleurocystis* Lloyd ex G. Cunn. 1956 (S) over *Matula* Massee 1888 (A)

The sexual morph of the type of *Aleurocystis*, *A. hakgallae* (as *Peniophora hakgallae*), was connected to the asexual morph *Matula poroniiforme*, type of *Matula*, by Petch (1926) and later accepted by Martin (1940) and Giraldo et al. (2017), thus the generic names *Aleurocystis* and *Matula* are synonyms. When he validated the name *Aleurocystis*, Cunningham (1956) included the generic name *Matula* as a synonym, and also listed *Artocreas poroniiforme* [as *poroniaeforme*] as a synonym of the type, *Aleurocystis hakgallae.* Cunningham (1956) also corrected the orthography from “*Aleurocystus*” that had been attributed to “McGinty” by Lloyd (1921). Under Art. F.8.1 both generic names *Aleurocytis* and *Matula* are legitimate. The basionyms for these species, namely *Corticium hakgallae* and *Artocreas poroniiforme,* were published in the same article (Berkeley and Broome 1875) and thus had equal priority. Cunningham (1956) placed *Artocreas poroniiforme* in synonymy of *Aleurocystis hakgallae,* thereby establishing priority for the species epithet, *hakgallae*. The only additional species in *Matula*, *M. rombellii*, is considered a synonym of *M. poroniiforme,* thus is *A. hakgallae* (Martin 1942). The only additional species of *Artocreas* is its lectotype, *A. micheneri*, now considered a synonym of *Scytinostroma artocreas* [see below under *Scytinostroma*]. The genus *Aleurocystis* has been widely used, includes four names (Ryvarden 1998; Hjorstam and Ryvarden 2000; Rajchenberg and Robledo 2005), and requires no name changes, thus *Aleurocystis* is recommended for protection.

One final note is that Massee (1888) published a new ordinal name, *Matulales* (as *Matuleae*)*,* typified by *Matula*, skipping over description of a family. Fortunately, priority of names only extends to the level of family (Art. 11.1).

### Protect *Armillaria* (Fr.) Staude 1857 (S) over *Rhizomorpha* Roth 1791 (A) and *Acurtis* Fr. 1849 (A/S)

*Armillaria* is a well-known genus of mushroom-forming fungi lectotypified by *A. mellea* (Clements and Shear 1931), the honey fungus, which is now recognized in a restricted sense within a complex amalgam of segregate species (Pegler 2000). The lectotype of *Rhizomorpha*, *R. fragilis,* represents rhizomorphs of *A. mellea* (Donk 1962), thus *Armillaria* and *Rhizomorpha* are synonyms. Donk (1962) provides a lengthy discussion of the lectotypification of *Rhizomorpha. Rhizomorpha* was accepted at the rank of genus in Fries (1821) and therefore is sanctioned, whereas *Agaricus* [unranked “tribus”] *Armillaria* is sanctioned only as an infrageneric name. Therefore, at the generic rank, *Armillaria* requires protection over *Rhizomorpha*. Although 117 names exist in *Rhizomorpha*, this generic name has been applied to the sterile rhizomorphs produced by different kinds of fungi not related to the type. For example, *Rhizomorpha hippotrichoides* is *Xylaria hippotrichoides* and *R. necatrix* is *Rosellinia necatrix*, both in *Ascomycota* (Kirk 2020). The generic name *Acurtis* was based upon *Clavaria gigantea,* which itself was based upon structures now recognized as the so called carpophoroids of *Entoloma abortivum*. For many years these carpophoroids were considered to be aborted, parasitized basidiomes of the *Entoloma* (Donk 1962; Watling 1974), but it has been shown that they are aborted basidiomes of *Armillaria* (Lindner Czederpiltz et al. 2001; Fukuda et al. 2003). Instead of threatening the name *Entoloma*, this oft debated generic name threatens the name *Armillaria*. Because *Armillaria* is a clearly circumscribed  and a well-known genus with species causing diseases of economic importance (Fox 2000), we recommend protection of the generic name *Armillaria*.

### Use *Arthrosporella* Singer 1970 (S) rather than *Nothoclavulina* Singer 1970 (A)

The monotypic generic names *Arthrosporella* and *Nothoclavulina* were published for the sexual and asexual morphs of the same species in the same publication (Singer 1970), specifically *A. ditopa* and *N. ditopa*, thus they are synonyms having equal priority. Here we designate *A. ditopa* as having priority. This species was re-described and discussed by Stalpers et al. (1991) and Baroni et al. (2007). Because *Arthrosporella* is more widely cited (GSS *Arthrosporella* = 24, *Nothoclavulina* = 11) and is typified by the mushroom-like sexual morph, we here designate *Arthrosporella* as having priority and recommend *Arthrosporella* for use.

### Protect *Asterophora* Ditmar 1809 (S) over *Ugola* Adans. 1763 (A) and use rather than *Nyctalis* Fr. 1825 (S)

The generic name *Asterophora* includes species that are parasitic on other mushrooms especially *Lactarius* and *Russula* (*Russulaceae*). Asexual morphs of species of *Asterophora* have been described in *Ugola* typified by *U. physaroides*, a synonym of *Asterophora physaroides*. Redhead and Seifert (2001a) unraveled and clarified the nomenclature of these generic names including *Nyctalis* as a synonym of *Asterophora*. They also proposed conservation of the name of the type of *Asterophora* as *A. lycoperdoides* over the earlier name *A. agaricoides* (Redhead and Seifert 2001b) and this was approved by the NCF (Gams 2004). Redhead and Seifert (2001a) recognized three species in *Ugola,* all of which have names in *Asterophora*. Redhead and Seifert (2001b) also recognized *A. lycoperdoides* and *A. physaroides* as the same species, thus *Asterophora* and *Ugola* are synonyms. Although *Ugola* has priority, this generic name is rarely used while *Asterophora* includes approximately 20 names and is widely used. For these reasons we recommend that *Asterophora* be protected over *Ugola* and be used instead of *Nyctalis*.

### Use *Athelia* Pers. 1818 (S) rather than *Fibularhizoctonia* G.C. Adams & Kropp 1996 (A)

The generic name *Athelia,* lectotypified by *A. epiphylla* by Donk (1949), includes about 40 species names and is widely used for saprobic, crustose, wood-inhabiting fungi as well as important plant pathogens (Jülich 1972). *Fibularhizoctonia*, typified by *F. carotae*, was described for the sclerotial-forming *Rhizoctonia carotae* (Rader 1948) and is recognized as the asexual morph of *Athelia arachnoidea*, a species that causes a cold-storage disease of carrots throughout temperate regions of the world (Adams and Kropp 1996). The two additional species of *Fibularhizoctonia*, sometimes misspelled ‘*Fibulorhizoctonia’*, are recognized in *Athelia* (De Vries et al. 2008; Kirk 2014). One species of *Athelia*, *A. rolfsii* (Curzi) C.C. Tu & Kimbr. 1978 (syn. *Sclerotium rolfsii* Sacc. 1911) is the scientific name for a soilborne pathogen that causes blight stem and root rot diseases of crop and nursery plants throughout the world (Punja 1985). *Athelia* is used much more commonly than *Fibularhizoctonia* (GSS *Athelia* = 5280, *Fibularhizoctonia* = 54). Given its priority, greater number of species, and widespread use, the generic name *Athelia* is recommended for use.

### Use *Bjerkandera* P. Karst. 1879 (S) over *Geotrichopsis* Tzean & Estey 1991 (A)

*Geotrichopsis* was introduced for *G. mycoparasitica*, a hyphomycete that produced thallic-arthric conidia and was isolated as a mycoparasite in a culture of an *Arthrobotrys* species. Because it had dolipore septa, it was considered to be the asexual state of a basidiomycete (Tzean and Estey 1991). It was described as new because it did not match the features in culture of various basidiomycetes known to produce asexual morphs, including *Polyporus adustus* (now *Bjerkandera adusta*). However, sequences of the ITS (MH862453) and LSU (MH874100) regions have been obtained from CBS 687.93, the ex-type culture of *G. mycoparasitica* (Vu et al. 2019), and the sequences have high BLAST matches to sequences identified in GenBank as *B. adusta* (ITS, 100% to several sequences including MF161298; LSU, 99.89% to KT305936). In a phylogenetic analysis of the combined ITS and LSU regions of a range of fungi isolated from *Prunus*, *G. mycoparasitica* CBS 687.93 fell within a well-supported clade otherwise comprised of *B. adusta* and *B. fumosa*, in a subclade with two sequences, one labelled *B. adusta* and the other *B.* cf. *adusta* (Bien and Damm 2020). Compared to sequences in the phylogeny of six species of *Bjerkandera* in Motato-Vásquez et al. (2020), which includes multiple sequences of each of *B. adusta* and *B. fumosa*, the ITS sequence from the ex-type culture of *G. mycoparasitica* has BLAST matches of 98.53–99.25% to sequences of *B. adusta*, 98.19–98.54% to sequences of *B. albocinerea* (the sister taxon to *B. adusta*), and 94.02–94.97% to sequences of *B. fumosa*. The sequence of *B. albocinerea* MH625420, which has the highest similarity, slightly overlapping with the range of similarity with *B. adusta*, is shorter, and lacks several characteristic bases that distinguish *B. albocinerea* from *B. adusta*. Consequently, *Geotrichopsis* should be considered a synonym of *Bjerkandera* and *G. mycoparasitica* placed in synonymy under *B. adusta*. Because *Geotrichopsis* has only been used for one species and is hardly mentioned in the literature (GSS *Bjerkandera* = 10,700, *Geotrichopsis* = 26), we recommend use of *Bjerkandera*.

### Protect *Botryobasidium* Donk 1931 (S) over *Acladium* Link 1809 (A), *Alysidium* Kunze 1817 (A), *Haplotrichum* Link 1824 (A), *Sporocephalium* Chevall. 1826 (A), *Physospora* Fr. 1835 (A), *Allescheriella* Henn. 1897 (A) and *Neoacladium* P.N. Singh & S.K. Singh 2019 (A)

The generic name *Botryobasidium* is typified by *B. subcoronatum*, while the holotype of *Haplotrichum* is *H. capitatum*. Holubová-Jechová (1976) recognized the generic name *Haplotrichum* for the asexual morphs of *Botryobasidium* “as a result of the conservation of the generic name *Oidium* for conidial states of *Erysiphe* …” (Partridge et al. 2001a) and this was accepted in a monographic account of *Botryobasidium* (Langer 1994). *Botryobasidium* includes species with or without *Haplotrichum* asexual morphs, clamp connections, smooth or ornamented basidiospores, and chlamydospores or cystidia, but excludes morphologically similar species that produce secondary spores or outgrowing clamps (Langer 1994). Partridge et al. (2001a, b, 2002) provided a comprehensive study of *Haplotrichum* in which *H. capitatum* is recognized as the asexual morph of *B. candicans*. *Haplotrichum capitatum* is based on *Acladium capitatum*, therefore, Rossman et al. (2016b) recombined this name as *Botryobasidium capitatum*. *Botryobasidium candicans* is now considered a synonym of *B. capitatum*. They also placed *Sporotrichum rubiginosum* in *Botryobasidium* as *B. rubiginosum*. The type of *Physospora*, *P. rubiginosa*, is also based on *Sporotrichum rubiginosum*, thus *Physospora* is a synonym of *Botryobasidium*. Donk (1962) reviewed the typification of *Physospora* concluding that Sumstine (1911) was the first to lectotypify this genus. The generic name *Physospora* was considered dubious by Donk (1962) and has been little used. *Acladium*, lectotypified by *A. conspersum*, now regarded as *B. conspersum*, is also congeneric with *Botryobasidium* and *Haplotrichum* as outlined by Holubová-Jechová (1976). Four species were originally included in *Acladium*. Both Clements and Shear (1931) and Hughes (1958) regarded *A. conspersum* as the type according to Donk (1962). The generic name *Alysidium* typified by *A. fulvum* has been considered a synonym of *Haplotrichum* (Holubová-Jechová 1980) as explained by Partridge et al. (2001a) who regarded *A. fulvum* as a synonym of the asexual morph of *B. aureum*. *Sporocephalium* was lectotypified with *S. capitatum* by Hughes (1958) who considered *Sporocephalium* a synonym of *Acladium.* Partridge et al. (2002) regarded *Acladium* to be a synonym of *Haplotrichum* with *Sporocephalium capitatum* as the asexual morph of *B. candicans*, now regarded as *B. capitatum*. Only four species of *Sporocephalium*, typified by *S. capitatum*, now *B. capitatum*, have been described, and this generic name is relatively obscure. Another generic name, *Allescheriella* is typified by *A. uredinoides*, now regarded as a synonym of *B. croceum* by Partridge et al. (2002), although Hughes (1951) recognized it as *B. fulvum*. Either way *Allescheriella* is a synonym of *Botryobasidium*. A recently described monotypic asexual morph generic name, *Neoacladium*, is here considered to be a synonym of *Botryobasidium* rather than sister to that genus (Hyde et al. 2019).

Despite the lack of a known asexual morph of *B. subcoronatum*, type of *Botryobasidium*, this species is considered to be congeneric with *Botryobasidium conspersum*, as shown by Binder and Hibbett (2002) and Larsson (2007), in which *H. conspersum*, *H. curtisii*, and *B. isabellinum* constitute a monophyletic group. Based on a nuclear ribosomal DNA large subunit (nLSU) analysis, Moncalvo et al. (2006) demonstrated that *Botryobasidium* included species with asexual morphs and smooth basidiospores (e. g. *B. candicans*, *B. conspersum*, *B. simile*,), species without asexual morphs and smooth basidiospores (e. g. *B. obtusisporum* and *B. vagum*), and species without an asexual morph and ornamented basidiospores (e. g. *B. isabellinum*) as well as the type *B. subcoronatum*. These species formed a well-supported monophyletic group as previously demonstrated by micromorphological and ultrastructural characters (Langer 1994; Langer and Langer 1998). Thus, *Botryobasidium*, *Acladium*, and *Haplotrichum*, as well as the lesser known *Alysidium*, *Sporocephalium*, *Physospora*, *Allescheriella*, and *Neoacladium*, are all synonyms. Although *Acladium*, *Haplotrichum*, *Physospora* and *Allescheriella* have priority, these names are less frequently cited than *Botryobasidium* (GSS *Botryobasidium* = 1010, *Acladium* = 884, *Alysidium* = 196, *Haplotrichum* = 206, *Sporocephalium* = 4, *Physospora* = 28, *Allescheriella* = 167). In addition, *Botryobasidium* includes a greater number of names, thus we recommend *Botryobasidium* for protection.

Five names in *Botryobasidium* are proposed for protection because they are widely used or already placed in *Botrybasidium*. In addition, 17 names described in *Haplotrichum* or other synonymous genera known to belong in *Botryobasidium* are re-combined in that genus here.

***Names proposed for protection:***

***Botryobasidium aureum*** Parmasto, Eesti N. S. V. Tead. Akad. Toimet., Biol. **14**: 220. 1965.

*Synonyms*: *Monilia aurea* J.F. Gmel., Syst. Nat., Edn 13 **2**(2): 1487. 1792.

*Trichoderma dubium* Pers., Syn. meth. fung. **1**: 233. 1801, nom. sanct. Fr., Syst. Mycol. **3**: 216. 1829.

Many additional earlier synonyms are listed in Kirk (2020).

***Botryobasidium conspersum*** J. Erikss., Symb. Bot. Upsal. **16**: 133. 1958.

*Type*: See Table 2.

*Synonyms to be protected over*: *Acladium conspersum* Link, Ges. Naturf. Freunde Berlin Mag. **3**: 11. 1809.

*Sporotrichum oosporum* Ehrenb., Sylv. mycol. berol. 22. 1818.

*Sporotrichum helvolum* Wallr., Fl. crypt. Germ. **2**: 280. 1833.

*Sporotrichum floccosum* Bres., Hedwigia **35**: 301. 1896.

*Rhinotrichum olivaceum* Bres., Fung. trident. **2**(14): 106. 1900.

*Rhinotrichum bicolor* Sumst., Mycologia **3**: 50. 1911.

*Rhinotrichum noblesiae* Sumst., Mycologia **29**: 250. 1937.

***Botryobasidium croceum*** Lentz, Mycopathol. Mycol. Appl. **32**: 6. 1967 “1966”.

*Type*: See Table 2.

*Synonyms to be protected over*: *Mucor croceus* Mont., Ann. Sci. Nat., Bot., sér. 2 **17**: 121. 1842.

*Gymnosporium fulvum* Berk. & M.A. Curtis, J. Linn. Soc., Bot. **10**: 355. 1868 “1869”.

*Rhinotrichum fulvum* (Berk. & M.A. Curtis) Berk. & M.A. Curtis, Grevillea **2**(19): 108. 1874.

*Allescheriella uredinioides* Henn., Hedwigia **36**: 244. 1897.

***Pellicularia lembospora*** D.P. Rogers, Farlowia **1**: 109. 1943 “1943–1944”.

*Synonyms*: *Botryobasidium lembosporum* (D.P. Rogers) Donk, Fungus **28**: 26. 1958.

*Hymenochaete tomentosa* Berk. & M.A. Curtis, J. Linn. Soc., Bot. **10**: 335. 1868 “1869”.

***Botryobasidium simile*** Hol.-Jech., Česká Mykol. **23**: 99. 1969.

*Synonyms*: *Oidium simile* Berk., J. Bot. (Hooker) **4**: 310. 1845.

*Monilia aureofulva* Cooke & Ellis, Grevillea **8**(no. 45): 12. 1879.

*Oidium biforme* Linder, Lloydia **5**: 188. 1942.

The names listed above for the sexual morph have older names that could be applied to the asexual morph. Because use of the oldest epithet would require a new combination that would displace a familiar and commonly used name, the five names in *Botryobasidium* listed above are proposed for protection. This synonymy is based primarily on Partridge et al. (2001a, b, 2002).

**New combinations:**

***Botryobasidium armeniacum*** (Berk. & M.A. Curtis) G. Langer, **comb. nov.**

MycoBank MB 837648

*Basionym*: *Rhinotrichum armeniacum* Berk. & M.A. Curtis, Grevillea **3**(27): 108. 1875.

***Botryobasidium caribense*** (Hol.-Jech.) G. Langer, **comb. nov.**

MycoBank MB 837649

*Basionym*: *Oidium caribense* Hol.-Jech., Ceská Mykol. **23**: 218. 1969.

***Botryobasidium elongatum*** (Linder) G. Langer, **comb. nov.**

MycoBank MB 837651

*Basionym*: *Oidium elongatum* Linder, Lloydia **5**: 191. 1942.

***Botryobasidium gracile*** (Hol.-Jech.) G. Langer, **comb. nov.**

MycoBank MB 837660

*Basionym*: *Haplotrichum gracile* Hol.-Jech., Ceská Mykol. **30**: 4. 1976.

***Botryobasidium indicum*** (P.H. Singh & S.K. Singh) R. Kirschner & G. Langer, **comb. nov.**

MycoBank MB 837873

*Basionym*: *Neoacladium indicum* P.N. Singh & S.K. Singh, Fungal Diversity **96**: 189. 2019.

***Botryobasidium laevisporum*** (Cooke) G. Langer, **comb. nov.**

MycoBank MB 837652

*Basionym*: *Zygodesmus laevisporus* Cooke, Grevillea **6**(40): 139. 1878.

***Botryobasidium magnisporum*** (Linder) G. Langer, **comb. nov.**

MycoBank MB 837653

*Basionym*: *Oidium magnisporum* Linder, Lloydia **5**: 179. 1942.

***Botryobasidium morganii*** (Linder) G. Langer, **comb. nov.**

MycoBank MB 837654

*Basionym*: *Oidium morganii* Linder, Lloydia **5**: 197. 1942.

***Botryobasidium ovalisporum*** (Linder) G. Langer, **comb. nov.**

MycoBank MB 837865

*Basionym*: *Oidium curtisii* var. *ovalisporum* Linder, Lloydia **5**: 204. 1942.

***Botryobasidium parmastoi*** (G. Langer) G. Langer, **comb. nov**.

MycoBank MB 837655

*Basionym*: *Haplotrichum parmastoi* G. Langer, Folia Cryptog. Estonica **33**: 63. 1998.

***Botryobasidium perseae*** (R.F. Castañeda) G. Langer, **comb. nov.**

MycoBan MB 837661

*Basionym*: *Haplotrichum perseae* R.F. Castañeda, Mycotaxon **59**: 449. 1996.

***Botryobasidium pulchrum*** (Berk.) G. Langer, **comb. nov.**

MycoBank MB 837662

*Basionym*: *Rhinotrichum pulchrum* Berk., J. Linn. Soc., Bot. **13**: 175. 1872 “1873”.

***Botryobasidium pulveraceum*** (Ellis) G. Langer, **comb. nov.**

MycoBank MB 837690

*Basionym*: *Monilia pulveracea* Ellis, Bull. Washburn Coll. Lab. Nat. Hist. **1**: 69. 1884.

***Botryobasidium ramosissimum*** (Berk. & M.A. Curtis) G. Langer, **comb. nov**.

MycoBank MB 837866

*Basionym*: *Rhinotrichum ramosissimum* Berk. & M.A. Curtis Grevillea **3**(27): 108. 1875.

***Botryobasidium sphaerosporum*** (Linder) G. Langer, **comb. nov.**

MycoBank MB 837867

*Basionym*: *Oidium sphaerosporum* Linder, Lloydia **5**: 200. 1942.

***Botryobasidium tenerum*** (Sumst.) G. Langer, **comb. nov**.

MycoBank MB 837691

*Basionym*: *Rhinotrichum tenerum* Sumst., Mycologia **3**: 51. 1911.

***Botryobasidium vesiculosum*** (Linder) G. Langer, **comb. nov.**

MycoBank MB 837693

*Basionym*: *Oidium vesiculosum* Linder, Lloydia **5**: 193. 1942.

### Use *Bullera* Derx 1930 (A) rather than *Bulleromyces* Boekhout & Á. Fonseca 1991 (S)

*Bulleromyces albus*, type of the monotypic generic name *Bulleromyces*, was described as the sexual state of *Bullera alba*, type of the generic name *Bullera* (Boekhout et al. 1991), thus *Bullera* and *Bulleromyces* are synonyms. *Bullera* is used more widely than *Bulleromyces* (GSS *Bullera* = 1230, *Bulleromyces* = 129). Given that *Bullera* has priority, currently includes over 60 species, and is widely used, we recommend the use of *Bullera*.

### Use *Chaetospermum* Sacc. 1892 (A) rather than *Efibulobasidium* K. Wells 1975 (S)

*Chaetospermum,* typified by *C. chaetosporum*, the name of the original illegitimate name C. *tubercularioides* (Smith and Ramsbottom 1914; Tangthirasunun et al. 2014), was placed in *Sebacinales* (Rungjindamai et al. 2008; Oberwinkler et al. 2014). A relationship of *Chaetospermum* to *Efibulobasidium* was shown by Wells and Bandoni (2001). Kirschner and Oberwinkler (2009) noted that conidia of *C. gossypinum* were associated with specimens of *E. albescens*, the type of *Efibulobasidium*, thus *Chaetospermum* and *Efibulobasidium* are most likely synonyms as accepted by Wells and Bandoni (2001) and Crous et al. (2014). In addition, *Chaetospermum camelliae* was studied by Kirschner et al. (2017) who placed its sexual morph in *Efibulobasidium*. Thirteen species have been accepted in *Chaetospermum* while only four names have been described in *Ebifulobasidium*, one of which has since been transferred to *Globulisebacina* (Oberwinkler et al. 2014) and two of which are invalid (Kirk 2020). Given its priority, the greater number of species, and more common usage (GSS *Chaetospermum* = 208, *Efibulobasidium* = 83), we recommend the use of *Chaetospermum*.

### Protect *Coprinellus* P. Karst. 1879 (S) over *Ozonium* Link 1809 (A)

The generic name *Coprinellus,* lectotypified by *C. deliquescens,* was resurrected for a segregate group of approximately 50 species previously placed in *Coprinus* (Redhead et al. 2001a). Many authors consider *Coprinellus domesticus* to be the sexual morph of *Ozonium auricomum*, type of the sanctioned generic name *Ozonium* (Plowright 1901; Buller 1924; Watling 1979). However, Cáceres et al. (2006) state that “it will probably never be possible to decide to which teleomorph belongs the type species *Ozonium auricomum*” and suggest referring to the asexual morph as “the *Ozonium* stage”. While one could determine the sexual morph of *O. auricomum* with an adequate holotype or lectotype and epitype with an ex-epitype culture, this is not necessary for the purpose of determining the synonymy of the genus *Ozonium* with *Coprinellus*. At the least *O. auricomum* is closely related to *C. domesticus*. Padamsee et al. (2008) demonstrated that *C. deliquescens* and *C. domesticus* are congeneric, thus *Coprinellus* and *Ozonium* are synonyms. Use of *Ozonium* for species now currently recognized as *Coprinellus* would not be tenable. Instead, the term “ozonium-like” should be used to describe the asexual morph of species of *Coprinellus* as suggested by Cáceres et al. (2006). In addition, the generic name *Coprinellus* is more widely used than *Ozonium* (GSS *Coprinellus* = 621, *Ozonium* = 98). Given the widespread use of *Coprinellus* and the many species placed in that genus, it is recommended that *Coprinellus* be protected.

The generic name *Ozonium* has also been used for a fungus unrelated to *O. auricomum,* namely the non-sporulating morph of the ascomycete *Phymatotrichopsis omnivora* (Shear) Hennebert 1973, often listed in the literature as *Ozonium omnivorum* (Shear 1907). Now placed in *Rhizinaceae (Pezizales), Phymatotrichopsis omnivora* is an ubiquitous, economically important plant pathogen that causes root rot of alfalfa, cotton, peanut, and pecan as well as diseases of approximately 2000 species of dicotyledonous plants (Marek et al. 2009).

### Protect *Coprinopsis* P. Karst. 1881 (S) over *Rhacophyllus* Berk. & Broome 1871 (A/S) and use rather than *Zerovaemyces* Gorovij (A/S) 1977 or *Hormographiella* Guarro & Gené 1992 (A)

The generic name *Coprinopsis,* lectotypified by *C. friesii*, is recognized for a group of species segregated from *Coprinus* and now includes over 100 species (Redhead et al. 2001a). Redhead et al. (2000) discussed in detail the debate over whether *Rhacophyllus* was based on an asexual or sexual morph and the history of the application of the name. Maniotis (1964) demonstrated that two morphs, a ‘*Coprinus*’ and *Rhacophyllus* represented one taxon. He named the sexual form *Coprinus clastophyllus*. Based upon morphological similarities to phylogenetetically classified taxa, Redhead et al. (2001a) considered *C. clastophyllus* and *C. friesii* to be congeneric and transferred *Coprinus clastophyllus* to *Coprinopsis*, thus *Coprinopsis* and *Rhacophyllus* are synonyms. While *Coprinopsis* is well known, *Rhacophyllus* has been rarely used (GSS *Coprinopsis* = 4530, *Rhacophyllus* = 45). The synonyms *Rhacophyllus lilacinus* and *Coprinopsis clastophylla* are currently both in use at a similar low frequency (GSS ca. 20 citations each, some of which are duplicates), and therefore there is no reason not to adopt the earlier name, which we do below. The generic name *Hormographiella,* typified by *H. aspergillata,* includes three species each isolated from animals or animal products, including humans (Guarro et al. 1992; Surmont et al. 2002). The relationship between the asexual morph *H. aspergillata* and the sexual morph *Coprinus cinereus* was determined by Gené et al. (1996) and *C. cinereus* was placed in *Coprinopsis* by Redhead et al. (2001a). Although *Coprinopsis friesii* falls in sect. *Alachuana* and *C. cinereus* in the separate *C. cinereus* clade, both species are confirmed as members of the genus *Coprinopsis* (Nagy et al. 2013a, b), thus *Coprinopsis* and *Hormographiella* are synonyms. *Coprinopsis* includes over 100 species and is widely known while only three names have been placed in *Hormographiella* and it is less commonly used (GSS *Hormographiella* = 302). *Zerovaemyces* was described with a single species, *Z. copriniformis,* by Gorovij (1977). This species name is probably synonymous with *Rhacophyllus lilacinus*. Given that *Coprinopsis* is widely known and includes many species, we recommend protection of *Coprinopsis* over *Rhacophyllus* and use of *Coprinopsis* rather than *Hormographiella* and *Zerovaemyces*.

A new family, *Zerovaemycetaceae*, was proposed by Gorovij (1977) and competes for use with *Psathyrellaceae* [see below under *Psathyrellaceae* for a discussion about this].

The advantages of using the generic name *Coprinopsis* over the earlier name *Pselliophora* P. Karst. 1879 were discussed by Redhead et al. (2001b). Ultimately, a proposal to conserve *Coprinopsis* over *Pselliophora* was accepted by the NCF (Gams 2005).

**New combination:**

***Coprinopsis lilacina*** (Berk. & Broome) Redhead, **comb. nov.**

Mycobank MB 838345

*Basionym: Rhacophyllus lilacinus* Berk. & Broome, J. Linn. Soc., Bot. **11:** 559. 1871.

*Synonyms*: *Coprinus clastophyllus* Maniotis, Amer. J. Bot. **51**: 491. 1964.

*Coprinopsis clastophylla* (Maniotis) Redhead et al., Taxon **50**: 227. 2001.

### Use *Cryptococcus* Vuill. 1901 (A) rather than *Filobasidiella* Kwon-Chung 1976 (S)

The generic name *Filobasidiella* was described by Kwon-Chung (1976) for the sexual morph of *Cryptococcus neoformans*, type of *Cryptococcus,* thus these generic names are synonyms. *Cryptococcus neoformans* and related species cause serious respiratory diseases of humans and animals that can be fatal for immunocompromised patients (May et al. 2016). More than 300 species had been placed in *Cryptococcus* but recently Liu et al. (2015) re-circumscribed *Cryptococcus* to include only ten species placing many names in other genera. All except one of the five names in *Filobasidiella* are now recognized in *Cryptococcus* (Kirk 2020; Liu et al. 2015). Given priority, its well-defined generic status, and widespread use especially for the medically important *C. neoformans*, we recommend the use of *Cryptococcus*.

### Use *Dacrymyces* Nees 1816 (S) rather than *Ditiola* Fr. 1822 (S), *Pionnotes* Fr. 1849 (A) or *Dacryoscyphus* R. Kirschner & Zhu L. Yang 2005 (A)

The generic name *Ditiola* Fr. 1822 was accepted over the pre-Friesian *Ditiola* P. Browne 1756, now regarded as *Schizophyllum,* as well as by Donk (1958) based on Nannfeldt (1947) and as accepted and explained by McNabb (1966). *Ditiola radicata* was designated as type of *Ditiola* by Brongniart (1824). This species was included in a study of *Dacrymyces* with *Ditiola radicata* grouping inside *Dacrymyces* (Shirouzu et al. 2013; Zamora and Ekman 2019), thus *Ditiola* is a synonym of *Dacrymyces*. The type specimen of the type of *Pionnotes*, *P. capitata* based on *Fusarium capitatum*, was re-examined by Seifert (2013) and determined to be *Dacrymyces chrysospermus*. Based on the phylogenetic trees presented by Shirouzu et al. (2007, 2009, 2013), *D. chrysospermus* is congeneric with the type of *Dacrymyces*, *D. stillatus*, thus *Pionnotes* is another synonym of *Dacrymyces*. *Pionnotes* has often been used in regard to species of *Fusarium,* while *Dacrymyces* includes over 100 names and is widely used. Phylogenetic studies of *Dacrymycetes* by Kirschner and Yang (2005), Shirouzu et al. (2009, 2013), and Zamora and Ekman (2020) demonstrated that the type of the asexually typified *Dacryoscyphus*, *D. chrysochilus*, grouped together with *Dacrymyces subarcticus* in *Dacrymyces*. *Dacrymyces* includes over  100 names while only three names have been placed in *Dacryoscyphus*. Unless the genus *Dacrymyces* is divided into many genera, *Dacryoscyphus* remains a synonym of *Dacrymyces*. Among these synonymous generic names, *Dacrymyces* is the most widely used (GSS *Dacrymyces* = 1580, *Ditiola* = 363, *Pionnotes* = 458, *Dacryoscyphus* = 16). Given its priority, greater number of names, and widespread use, we recommend the use of *Dacrymyces*.

### Use *Deconica* (W.G. Sm.) P. Karst. 1879 (S) rather than *Pseudohelicomyces* Garnica & E. Valenz. 2000 (A)

*Pseudohelicomyces albus*, type of the monotypic generic name *Pseudohelicomyces*, was described for the asexual morph of *Psilocybe merdaria* (Valenzuela and Garnica 2000), now regarded as *Deconica merdaria* (Noordeloos 2009). Since then, the generic name *Psilocybe* has been proposed for conservation with a new type, *P. semilanceata*, in order to include species that produce psilocybin (Redhead et al. 2007); this proposal was accepted (Norvell 2010) and is included in Appendix III of the ICN (Wiersema et al. 2020). Thus, *P. merdaria* and other non-hallucinogenic species previously recognized in *Psilocybe* have been shown to fall outside of the group that includes the conserved type of *Psilocybe* (Moncalvo et al. 2002). Although the type of *Deconica*, *D. bullacea*, has not been sequenced, it is accepted that *D. bullacea* and *D. merdaria* are congeneric (Noordeloos 2009), thus *Deconica* and *Pseudohelicomyces* are synonyms. *Deconica* includes 59 names and is widely used (GSS = 400) while monotypic *Pseudohelicomyces* remains an obscure name (GSS = 21). Given these reasons and based on priority, we recommend the use of *Deconica*.

### Protect *Dendrocollybia* R.H. Petersen & Redhead 2001 (S) over *Tilachlidiopsis* Keissl. 1924 (A) and *Sclerostilbum* Povah 1932 (A)

The generic name *Dendrocollybia* is typified by *D. racemosa,* a species previously placed in *Collybia* (Hughes et al. 2001). The asexual morph of *D. racemosa* has been recognized in *Tilachlidiopsis* typified by *T. racemosa* of which *Sclerostilbum septentrionale*, the type of the monotypic generic name *Sclerostilbum*, was considered a synonym (Stalpers et al. 1991; Hughes et al. 2001). Thus, the types of the latter two generic names are synonyms of *D. racemosa* and these three generic names are synonyms. *Tilachlidiopsis* has included diverse species in the *Ascomycota* and *Basidiomycota* (Stalpers et al. 1991) and is rarely used for the latter. Hofstetter et al. (2014) showed that *Dendrocollybia* should be recognized as a distinct genus. Although *Tilachlidiopsis* has been used more than *Dendrocollybia*, many of those references are to names now placed in the ascomycete genus *Ophiocordyceps* (GSS *Dendrocollybia* = 85, *D. racemosa* = 61, *Tilachlidiopsis* = 89, *T. racemosa* = 23, *Sclerostilbum* = 45). Given the widespread use of *Dendrocollybia* and its distinct morphology, we recommend that *Dendrocollybia* be protected over *Tilachlidiopsis* and *Sclerostilbum*.

### Protect *Diacanthodes* Singer 1962 (S) over *Bornetina* L. Mangin & Viala 1903 (A)

The genus *Diacanthodes,* typified by *D. novoguineensis,* includes six species with two varieties of tropical fungi (Robledo et al. 2020). The asexual morph of *D. novoguineensis* was determined to be *Bornetina corium* in the monotypic genus *Bornetina* (Fidalgo 1962a, b), thus *Diacanthodes* and *Bornetina* are synonyms. *Diacanthodes novoguineensis* causes a root disease known as phthiriasis of coffee and other tropical hosts (Rajchenberg and Robledo 2013). *Diacanthodes* is used more frequently (GSS = 118) and includes more species than *Bornetina* (GSS = 70), thus we recommend *Diacanthodes* for protection.

### Use *Ditangium* P. Karst. 1867 (A) rather than *Craterocolla* Bref. 1888 (S) or *Poroidea* Göttinger ex G. Winter 1885 (A)

The connection between *Craterocolla, Ditangium* and *Poroidea* was reviewed by Donk (1962, 1966) who concluded that they were sexual and asexual morphs of the same species. *Craterocolla,* typified by *C. cerasi,* was confirmed as a distinct genus in *Sebacinales* by Weiss and Oberwinkler (2001). The generic name *Ditangium* is typified by *D. insigne* of which *Craterocolla cerasi* is a synonym (Donk 1962; Nag Raj 1978; Malysheva et al. 2019). Nag Raj (1978) included the monotypic *Poroidea,* typified by *P. pithyophila,* as a synonym of the asexual morph of *Craterocolla cerasi*. Thus, *Craterocolla, Ditangium,* and *Poroidea* are synonyms. Although *Craterocolla* is more widely used than *Ditangium* (GSS *Craterocolla* = 127, *Ditangium* = 34, *Poroidea* = 8), *Ditangium* has priority and was recommended for use by Malysheva et al. (2019), and we follow their recommendation.

### Protect *Echinoporia* Ryvarden 1980 (S) over *Echinodia* Pat. 1918 (A)

The generic name *Echinoporia,* typified by *E. hydnophora,* was described as the sexual morph of *Echinodia theobromae*, the type of the monotypic genus *Echinodia* (Ryvarden and Johansen 1980; Ryvarden 1983), thus *Echinoporia* and *Echinodia* are synonyms. *Echinoporia* includes three species and is more commonly used than *Echinodia* (GSS *Echinoporia* = 66, *Echinodia* = 15). Because it has a greater number of species and has been used in the recent literature (Motato-Vasquez et al. 2015), we recommend the protection of *Echinoporia*.

### Use *Femsjonia* Fr. 1849 (S) rather than *Cerinosterus* R.T. Moore 1987 (A)

The genus *Femsjonia* is typified by *F. luteoalba* for which *Exidia peziziformis* provides an earlier epithet, thus this species is regarded as *F. peziziformis* (McNabb 1965). The putative asexual morph of *F. peziziformis* (as *F. luteoalba*) was named by de Hoog (1974) as *Sporothrix luteoalba*, and the connection was reiterated by Maekawa (1987) and Middlehoven et al. (2000). When *Cerinosterus* was described, it was typified by *S. luteoalba*, which was erroneously interpreted as the asexual morph of a *Cerinomyces* sp. (Moore 1987). We confirm the relationship of *Cerinosterus luteoalbus* with *F. peziziformis* based on the 100% match of a BLAST search of an ITS sequence (MH856312) from an ex-paratype strain of *C. luteoalbus* (CBS 208.48) with *F. peziziformis.* Thus, *Cerinosterus* and *Femsjonia* are synonyms. *Femsjonia* includes 12 names while only two names have been placed in *Cerinosterus,* one of which, *C. cyanescens,* is now placed in *Quambalaria* (de Beer et al. 2006). Shirouzu et al. (2009, 2017) demonstrated that *F. peziziformis* groups outside of *Dacrymyces* and this was confirmed by Zamora and Ekman (2019) who placed *Femsjonia* in the *Dacrymycetaceae*. Given its priority, the greater number of names, and greater use (GSS *Femsjonia* = 175, *Cerinosterus* = 71), we recommend the use of *Femsjonia*.

### Use *Fistulina* Bull. 1791 (S) rather than *Confistulina* Stalpers 1983 (A)

The asexual morph of *Fistulina hepatica*, type of *Fistulina,* was described in the monotypic genus *Confistulina* typified by *C. hepatica* (Stalpers and Vlug 1983), thus these generic names are synonyms. *Fistulina* currently includes 21 names and is widely used (GSS *Fistulina* = 2900, *Confistulina* = 23). Given its priority, greater number of species, and its widespread use, *Fistulina* is recommended for use.

### Use *Heteroacanthella* Oberw. 1990 (S) rather than *Acanthellorhiza* P. Roberts 1999 (A)

The monotypic generic name *Acanthellorhiza* is typified by *A. globulifera*, which is the asexual morph of *Heteroacanthella acanthophysa* (Roberts 1999). *Heteroacanthella* is typified by *H. variabilis* (Oberwinkler et al. 1990). Zamora et al. (2014) regarded *H. acanthophysa* and *H. variabilis* as congeneric and added a third species. *Heteroacanthella* is more widely used than *Acanthellorhiza* (GSS *Heteroacanthella* = 35, *Acanthellorhiza* = 4). *Heteroacanthella* has priority and includes three species, while *Acanthellorhiza* is monotypic and remains little known, thus we recommend the use of *Heteroacanthella*.

### Use *Heterobasidion* Bref. 1888 (S) rather than *Spiniger* Stalpers 1974 (A)

The holotype of *Heterobasidion, H. annosum*, is the sexual morph of the type of *Spiniger*, *S*. *meineckellus* (Stalpers 1974), thus these generic names are synonyms. *Heterobasidion annosum,* often reported as its synonym *Fomes annosus*, is the cause of a serious tree disease resulting in root and butt rot of pine trees in Eurasia, although previously this name had been used to represent a species complex occurring in temperate coniferous forests throughout the world (Ostrosina and Garbelotto 2010; Pegler and Waterston 1968). A number of segregate species have now been described in *Heterobasidion* (Ostrosina and Garbelotto 2010). *Heterobasidion* includes about 30 species, has priority, and is well known (GSS *Heterobasidion* = 14,200, *Spiniger* = 5700), while *Spiniger* includes only one additional species and remains relatively obscure, thus we recommend the use of *Heterobasidion*.

### Use *Hohenbuehelia* Schultzer 1866 (S) rather than *Nematoctonus* Drechsler 1941 (A)

The generic synonymy of *Hohenbuehelia,* typified by *H. petaloides,* and *Nematoctonus,* typified by *N. tylosporus,* was proven by Koziak et al. (2007) in which they showed that *Hohenbuehelia-Nematoctonus* including their types formed a monophyletic clade in *Pleurotaceae*. Donk (1962) was the first to designate *N. tylosporus* as the type of *Nematoctonus* stating that he chose this as the best species to represent the genus. Thorn (2013) made the nomenclatural changes consistent with the recognition of the use of *Hohenbuehelia* rather than *Nematoctonus*. *Hohenbuehelia* produces a mushroom-like sexual morph and is more widely used than the nematode-trapping asexual morph represented by *Nematoctonus* (GSS *Hohenbuehelia* = 1500, *Nematoctonus* = 564). Given that *Hohenbuehelia* has priority and includes 182 names while *Nematoctonus* includes only 16 names and in agreement with Thorn (2013), we recommend *Hohenbuehelia* for use.

### Use *Leucocoprinus* Pat. 1888 (S) rather than *Attamyces* Kreisel 1972 (A)

The monotypic generic name *Attamyces* is typified by *A. bromatificus*, a bromatia-forming fungus associated with attine or leaf-cutter ant nests in the western hemisphere (Kreisel 1972). *Attamyces bromatificus* was considered to refer to an asexual morph of *Leucoagaricus gongylophorus* (Chapela et al. 1994; North et al. 1997). Ortiz et al. (2008) analyzed sequences from *L. gongylophorus* and discussed the taxon under *Leucoagaricus*, although much earlier the combination *Leucocoprinus gongylophorus* had been introduced by Heim (1957). Controversy exists over the use of *Leucocoprinus* and *Leucoagaricus.* The older name *Leucocoprinus* is lectotypified by *L. cepistipes,* while confusion had existed over the type of *Leucoagaricus,* first validly published by Singer (1948) because he suggested as type an invalid name while including two other validly published names. This dilemma was resolved by Redhead (2016) who lectotypified the generic name *Leucoagaricus* by *L. rubrotinctus*, thus overriding the classification of *Leucocoprinus americanus* as *Leucoagaricus americanus* by Vellinga (2000). Phylogenetic analyses of DNA sequence data place numerous species of *Leucocoprinus* and *Leucoagaricus*, including their types, in the same clade with an intermixing of species from each genus (Vellinga et al. 2011). Despite the evident synonymy of *Leucoagaricus* with *Leucocoprinus*, novel species continue to be described in both genera (Ge et al. 2015) pending a comprehensive treatment of the group. For the purpose of placing *Attamyces*, we treat *Leucocoprinus* and *Leucoagaricus* as synonyms with *Leucocoprinus* having priority. At present, *Attamyces* should be regarded as a synonym of *Leucocoprinus*. Given its greater number of names and widespread use, we recommend that *Leucocoprinus* be used rather than *Attamyces*.

*Coccobotrys* is another generic name that has been mentioned as a synonym of members of *Agaricaceae* such as *Leucoagaricus*. When introducing *Coccobotrys*, typified by *C. xylophilus*, Boudier and Patouillard (1900) noted that the specimen of the type from France differed in some respects from the characteristics of the original description of the basionym *Cenococcum xylophilum* Fr. 1829. Van Bambeke (1900) reported further material from Belgium, which he regarded as conspecific with the French specimen observed by Boudier and Patouillard (1900). Van Bambeke (1900) considered *Coccobotrys xylophilus* to be an asexual morph of *Lepiota meleagris* (now *Leucoagaricus meleagris*). According to Else C. Vellinga (pers. comm.), the material examined by Boudier and Patouillard (1900) and van Bambeke (1900) is not conspecific with the original collection of *Cenococcum xylophilum* from Russia. Therefore, the recognition of *Coccobotrys* as a lepiotaceous fungus is based on a misapplication. The identity of the type of *Coccobotrys* is unknown. A second species of *Coccobotrys, C. chilensis,* was transferred to *Leucoagaricus* by Ruiz and Molinari-Novoa (2016). Note also that *Coccobotrys* could be confused with the later homonym *Coccobotrys* R. Chodat 1913, a name applied to a group of algae.

### Use *Marchandiomyces* Diederich. & D. Hawksw. 1990 (A) rather than *Marchandiopsis* Ghobad-Nejhad & Hallenb. 2010 (S)

Ghobad-Nejhad et al. (2010) demonstrated that the species originally described as *Laeticorticium quercinum* falls within a clade also containing *Marchandiomyces corallinus*, an asexual morph that is the type of *Marchandiomyces*. They chose to place the two species in the same genus, but under the rules of nomenclature at the time, they could not take up *Marchandiomyces* for the basidiospore-producing *L. quercinum*. Therefore, they introduced *Marchandiopsis*, typified by *L. quercinum*. With the move to one fungus-one name, Hawksworth and Henrici (2015) argued that *Marchandiopsis* had rarely been used in comparison to *Marchandiomyces* and they proposed to take up the latter name. They made the necessary new combination, *Marchandiomyces quercinus*. Diederich et al. (2018) transferred three other species originally described in *Marchandiomyces* to *Laetisaria*, placed *Marchandiomyces aurantiacus* in *Erythricium*, and retained three species in *Marchandiomyces*, including *M. quercinus* and *M. corallinus*. Given current usage of *Marchandiomyces* (GSS *Marchandiomyces* = 404, *Marchandiopsis* = 9) and in agreement with recent authors, we recommend that *Marchandiomyces* be used.

### Use *Mycena* (Pers.) Roussel (S) 1806 rather than *Decapitatus* Redhead & Seifert (A) 2000

The generic name *Mycena,* typified by *M. galericulata,* is used for over 2000 species of small mushrooms, while the monotypic *Decapitatus,* typified by *D. flavidus* based on *Stilbum flavidum,* was used for the asexual morph of *Mycena citricolor* (Seifert 1985; Redhead et al. 2000). Several authors have considered *M. citricolor* and *M. galericulata* to be congeneric (Bermudes et al. 1991; Desjardin et al. 2007), thus *Mycena* and *Decapitatus* are synonyms. *Mycena* is more widely used than *Decapitatus* (GSS *Mycena* = 11,400, *Decapitatus* = 243). Given the widespread use of *Mycena,* the high number of species, and its priority, we recommend *Mycena* for use when *Mycena* is adopted wth a broad concept.

### Use *Myxarium* Wallr. 1833 (S) rather than *Hyaloria* Möller 1895 (S) or *Helicomyxa* R. Kirschner & Chee J. Chen 2004 (A)

*Myxarium nucleatum*, the type of *Myxarium*, and *M. mesonucleatum* were shown to form a strongly supported clade with *Hyaloria pilacre*, type of the generic name *Hyaloria,* by Kirschner and Chen (2004), thus *Myxarium* and *Hyaloria* are synonyms. They also showed that the type of the monotypic generic name *Helicomyxa*, *H. everhartioides*, is closely related to *Myxarium*. The small conidiomata with conidia of *M. tremelloides* (as *Exidia tremelloides*) are similar to those of *H. everhartioides* suggesting that these names are synonyms or at least closely related. Thus, the available data suggest that *Helicomyxa, Hyaloria,* and *Myxarium* are synonyms. Given the greater number of species, greater use (GSS *Myxarium* = 213, *Hyaloria* = 53, *Helicomyxa* = 22), and priority, we recommend the use of *Myxarium*.

***New combinations:***

***Myxarium everhartioides*** (R. Kirschner & Chee J. Chen) R. Kirschner, **comb. nov.**

MycoBank MB 837868

*Basionym*: *Helicomyxa everhartioides* R. Kirschner & Chee J. Chen, Stud. Mycol. **50**: 339. 2004.

***Myxarium pilacre*** (Möller) R. Kirschner, **comb. nov.**

MycoBank MB 837869

*Basionym*: *Hyaloria pilacre* Möller, Bot. Mitt. Tropen **8**: 173. 1895.

### Use *Necator* Massee 1898 (A) over *Upasia* Harsojo-Tjokrosoedarmo & Rifai 1992 (A/S)

*Necator decretus* was described as a destructive parasite of young coffee branches in Malaysia (Massee 1898). Rant (1911) connected *Necator* to an unnamed *Corticium* and later Rant (1912) connected it to *Corticium javanicum* and *C. salmonicolor* (Petch 1912). Most recently the fungus in all stages has been classified as *Erythricium salmonicolor* (e. g. Moraes et al. 2006) but also has been shown to be an independent sister taxon to *Erythricium* (Roux and Coetzee 2005; Ghobad-Nejhad et al. 2010; Diederich et al. 2011). In an overlooked publication, Harsojo-Tjokrosoedarmo (1992, 1995) proposed a new genus *Upasia* for *E. salmonico*lor. Recognized as a genus separate from *Erythricium*, *Necator* has priority and is here recommended for use. A new combination in *Necator* is required and proposed here.

*Erythricium,* typified by *E. laetum*, is considered to be a separate genus that includes as a synonym *Marchandiobasidium,* typified by *M. aurantiacum* now recognized as *Erythricium aurantiacum* (Hawksworth and Henrici 2015; Diederich et al. 2018))*. Erythricium* and its synonym *Marchandiobasidium* are sexual morph names.

***Necator salmonicolor*** (Berk. & Broome) K.H. Larss., Redhead, & T.W. May, **comb. nov.**

MycoBank MB 838387

*Basionym*: *Corticium salmonicolor* Berk. & Broome, J. Linn. Soc., Bot. **14**(74): 71. 1873 "1875".

### Protect *Neolentinus* Redhead & Ginns 1985 (S) over *Digitellus* Paulet 1793 (A/S)

*Digitellus humanus*, the lectotype of *Digitellus,* is presumed to be based upon the aborted basidiomes of *Neolentinus*, most probably *N. lepideus* (cf. Donk 1962; Redhead and Ginns 1985), which typically do not form expanded pilei but the sterile stipes often become multibranched in complete darkness, such as on supporting timbers in abandoned mine shafts (Vlasenko et al. 2017). Hence, the generic name and species epithet refer to a hand-like form, which have also been named *Neolentinus lepideus* f. *ceratoides* based upon *Ramaria ceratoides*. The validity of the Paulet names and the date of Paulet’s 1793 publication have been questioned because of its production during the French revolution and conflicting accounts of its distribution. Presuming the name *Digitellus* published as genus XVI in his index is valid and noting its obscurity, when *Neolentinus* typified by *N. kauffmanii* includes *N. lepideus*, we recommend that the name *Neolentinus* should be protected. This is subject to resolution of a proposal to list Paulet’s publication as a “Suppressed Work” (Parra et al. 2015).

### Use *Oliveonia* Donk 1958 (S) rather than *Oliveorhiza* P. Roberts 1998 (A)

The generic name *Oliveonia,* typified by *O. fibrillosa,* was established to replace the later homonym *Heteromyces* Olive 1957 non Müll. Arg. 1889 (Donk 1958). Although regarded as related to *Ceratobasidium*, *Oliveonia* has been shown to be distinct including five species (Kotiranta and Saarenoksa 2005). *Oliveorhiza anapauxilla*, the type and only species of *Oliveorhiza,* was described for the asexual morph of *Oliveonia pauxilla* (Roberts 1998). *Oliveonia fibrillosa* and *O. pauxilla* are congeneric (Kotiranta and Saarenoksa 2005; Roberts 1998), thus *Oliveonia* and *Oliveorhiza* are synonyms. *Oliveonia* has been more widely used than *Oliveorhiza* (GSS *Oliveonia* = 107, *Oliveorhiza* = 6). Given its priority, greater number of species, and widespread use, we recommend the use of *Oliveonia*.

### Use *Pleurotus* (Fr.) P. Kumm. 1871 (S) rather than *Antromycopsis* Pat. & Trab. 1897 (A)

The generic name *Pleurotus* is applied to a number of mushroom-forming fungi including the type, *P. ostreatus,* the oyster mushroom, one of the most commonly cultivated edible fungi in the world, and *P. eryngii* (DC.) Quél. 1872, the widely consumed king oyster mushroom (Zervakis et al. 2004). Species of *Pleurotus* are also used industrially to break down aromatic hydrocarbons (Adenipekun et al. 2015); in addition, they can produce toxic compounds that paralyze nematodes. The generic name *Pleurotus* has already been conserved over six earlier generic names (Wiersema et al. 2020). Miller Jr. (1969) described *P. cystidiosus* for the sexual morph of *Antromycopsis broussonetiae*, type of the generic name *Antromycopsis*. *Pleurotus ostreatus* and *P. cystidiosus* were shown to be congeneric by Gonzalez and Labarère (2000), thus *Pleurotus* and *Antromycopsis* are synonyms. The genus *Pleurotus* includes over 500 species names; of these, a few dozen have been well-studied and are nematophagous (Thorn and Barron 1984; Barron and Thorn 1987), whereas the name *Antromycopsis* is rarely used and includes only 19 names (GSS *Pleurotus* = 132,000, *Antromycopsis* = 175). Considering the widespread use of *Pleurotus,* its numerous species, its economic importance, and its priority, we recommended the use of *Pleurotus*.

### Use *Polyporus* P. Micheli ex Adans. 1763 (S) rather than *Mycelithe* Gasp. 1841 (A)

The monotypic generic name *Mycelithe,* typified by *M. fungifera,* accommodates sclerotia of *Polyporus tuberaster*, lectotype of *Polyporus.* Donk (1962) provides one of the few references to the obscure generic name *Mycelithe.* Based on the original well-illustrated publication, Gasparrini (1842) concluded that *M. fungifera* refers to sclerotia of a species in *Polyporus*, thus these two generic names are synonyms. *Polyporus* is a widely used name with over 3000 names described in this genus while the monotypic *Mycelithe* is uncommonly used (GSS *Polyporus* = 30,100, *Mycelithe* = 7). Given its widespread use and priority as well as the numerous described species, we recommend the use of *Polyporus*.

Controversy exists regarding the typification of the sanctioned name *Polyporus*. The earliest non-mechanical lectotypification was by Clements and Shear (1931) who selected *P. brumalis* for “*Polyporus* (Mich.) Fr. Epicr. 427 1838”. However, Fries (1838) considered the generic name to link back to Fries (1821) and consequently Donk (1933) selected *P. tuberaster* as lectotype from species included by Fries (1821). For the moment, we follow Donk (1933) and current usage by indicating *P. tuberaster* as type of *Polyporus*, but we note that formal conservation with a conserved type may be required.

### Protect *Postia* Fr. 1874 (S) over *Ptychogaster* Corda 1838 (A)

*Postia,* lectotypified by *P. lactea,* is a well-defined genus as recently circumscribed by Pildain and Rajchenberg (2013) and Shen et al. (2015, 2019). Walker (1996) provided an extensive discussion of the validity of the publication of the generic name *Postia.* The type of *Ptychogaster*, *P. albus*, was placed in *Postia* as *P. ptychogaster* by Knudsen and Hansen (1996) and is widely reported (Shen et al. 2015; Vampola et al. 2014; Vizzini and Zotti 2008). Given that *Postia lactea* and *P. ptychogaster* are congeneric, as demonstrated by Shen et al. (2015), then *Postia* and *Ptychogaster* are synonyms. Most names in *Ptychogaster* are now recognized in *Postia* or other related genera (Stalpers 2000). *Postia* is the most widely used of these two names (GSS *Postia* = 7950, *Ptychogaster* = 617), thus we recommend protection and use of the generic name *Postia*.

Two older synonyms are known for *Postia ptychogaster* based on *Polyporus ptychogaster* but neither of these synonyms listed below are widely used. This synonymy has been supported by Shevchenko (2018) and Stalpers (2000), thus we propose the basionym *Polyporus ptychogaster* for protection:

***Polyporus ptychogaster*** F. Ludw., Z. Gesammten Naturwiss. (Halle) **3**: 424. 1880.

Lectotype: See Table 2.

*Accepted name*: *Postia ptychogaster* (F. Ludw.) Vesterh., Nordic J. Bot. **16**: 213. 1996.

*Rejected synonyms*: *Ptychogaster albus* Corda, Icon. fung*.* **2**: 24. 1838.

*Trichoderma fuliginoides* Pers., Syn. meth. fung*.* **1**: 231. 1801.

### Protect *Psathyrellaceae* Vilgalys et al. 2001 over *Zerovaemycetaceae* Gorovij 1977

*Zerovaemyces* is regarded as a synonym of *Coprinopsis* with the type of *Zerovaemyces, Z. copriniformis,* considered a synonym of *Rhacophyllus lilacinus*, now *Coprinopsis lilacina* as discussed above under *Coprinopsis*. Gorovij (1979) published a new class name*, Loculomycetes*. Redhead et al. (2000) argued that *Zerovaemyces* and *Zerovaemycetaceae*, the latter introduced by Gorovij (1979), were asexual morphs and using the Code in force in 2000–2001, Redhead et al. (2001a) elevated *Coprinaceae* subfam. *Psathyrelloideae* Singer to family level with priority based upon the date 2001. *Coprinopsis* (syn. *Zerovaemyces*) is classified in the *Psathyrellaceae* in modern phylogenetic analyses, thus *Zerovaemycetaceae* is a synonym of *Psathyrellaceae*. *Psathyrellaceae* appears in over 1500 GS records while *Zerovaemycetaceae* appears in 3 GS hits, two of which are Redhead et al. (2000, 2001a). The name *Psathyrellaceae* is adopted in modern phylogenetic analyses and in mushroom books. It would be disruptive to switch to a family name used so infrequently and based upon synonymized taxon names, thus we propose the name *Psathyrellaceae* for protection.

### Use *Rhizoctonia* DC. 1815 (A) rather than *Thanatephorus* Donk 1956 (S)

The generic name *Rhizoctonia* has been conserved with *R. solani* as the conserved type and *R. solani* is formally conserved against *R. napaeae* (Stalpers et al. 1998; Wiersema et al. 2020). *Rhizoctonia* includes many species, especially ones causing plant diseases (Ajayi-Oyetunde and Bradley 2018; Oberwinkler et al. 2013). Some names in *Rhizoctonia* have been shown to be ascomycetous fungi and removed to *Ascorhizoctonia* and other genera (Yang and Korf 1985) while others have been transferred to *Waitea*. By far the most important species is the ubiquitous type *R. solani,* which has been divided into anastomosis groups and causes diseases such as root rot, damping off in seedlings, and leaf and fruit rot of many plant hosts (Gonzales et al. 2016). The sexual morph of *R. solani* was described as *Thanatephorus cucumeris*, type of the genus *Thanatephorus* (Roberts 1999), thus *Rhizoctonia* and *Thanatephorus* are synonyms. The decision to use *Rhizoctonia* or *Thanatephorus* is a difficult one; however, *Rhizoctonia* is used more commonly albeit in a broad sense and has priority (GSS *Rhizoctonia* = 22,400, *Thanatephorus* = 1930), thus we recommend the use of *Rhizoctonia*.

See below in regard to species of *Rhizoctonia* that belong to *Waitea*.

### Protect *Riopa* D.A. Reid 1969 (S) over *Sporotrichum* Link 1809 (A)

The generic name *Riopa,* previously considered a synonym of *Ceriporia,* is typified by *R. davidii*, which is regarded as a synonym of the older name *R. metamorphosa* by Miettinen et al. (2016). Based on the concept of Miettinen et al. (2016), the genus includes one additional species *R. pudens*. The typification of *Sporotrichum* is complicated. *Sporotrichum* has been cited as having various types over time, and the type accepted by recent authors, *S. aureum* Link 1809 (Hughes 1958; Donk 1962; Stalpers 1984; Miettinen et al. 2016), is a homonym of the sanctioned name *S. aureum* (Pers.) Fr. 1832 and thus not available without conservation. Stalpers (1984) and Miettinen et al. (2016) considered *S. aureum* Link 1809 synonymous with *S. aurantiacum* Fr.*,* which is the asexual morph of *R. metamorphosa*. Although more than 300 names have been placed in *Sporotrichum*, the majority of these names belong in *Ascomycotina*. Stalpers (1984) accepted three species in *Sporotrichum*, each of which has been shown to be phylogenetically distinct, i. e. not congeneric (Miettinen et al. 2016), thus *Sporotrichum* now includes only the type, *S. aurantiacum* Fr. 1832, a sanctioned, legitimate, later homonym of *S. aurantiacum* Grev. 1822 based on *Mucor aurantius* Bull. 1791. We follow Miettinen et al. (2016) and recommend *Riopa* for protection over *Sporotrichum*; in addition, we recommend the protection of *R. metamorphosa* over *S. aurantiacum* Fr.

***Name proposed for protection*** *:*

***Riopa metamorphosa*** (Fuckel) Miettinen & Spirin, MycoKeys **17**: 27. 2016.

*Basionym*: *Polyporus metamorphosus* Fuckel, Jahrb. Nassauischen Vereins Naturk. **27-28**: 87. 1874.

*Type:* see Table 2.

*Synonyms to be protected over*: *Mucor aurantius* Bull., Hist. Champ. France **1**: 103. V. 1791.

*Sporotrichum aurantiacum* Fr., Syst. Mycol. **3**: 423. 1832, nom. sanct., non Grev., Mem. Wern. Nat. Hist. Soc. **4**: 67. 1822.

### Protect *Scytinostroma* Donk 1956 (S) over *Michenera* Berk. & M.A. Curtis 1869 (A), *Artocreas* Berk. & Broome 1873 (A/S), and *Stereofomes* Rick 1928 (S) and use rather than *Licrostroma* P.A. Lemke 1964 (S)

The sexual morph of *Michenera,* typified by *M. artocreas,* was described as *Licrostroma,* typified by *L. subgiganteum,* by Lemke (1964). Lyman (1907) had earlier demonstrated that cultures derived from the basidiospores of what he called *Corticium subgiganteum,* the basionym of *L. subgiganteum*, produced what he called *Michenera* spores, but he was unable to induce the cultures to form hymenia and basidiospores. Molecular data place *L. subgiganteum* with *Scytinostroma aluta* and *S. portentosum*, the type of *Scytinostroma* (Giraldo et al. 2017; K-H Larsson pers. comm. 2017) and with *S. caudisporum* (Leal-Dutra et al. 2020). Thus, *Scytinostroma*, *Michenera,* and *Licrostrom*a are synonyms. Giraldo et al. (2017) recognized the synonymy of *Licrostroma* with *Michenera* but chose to use *Licrostroma* despite the priority of *Michenera*. Conversely, Liu et al. (2019) recognized *Michenera* including a new species; their phylogeny placed both the type and the new species of *Michenera* in a clade with the type of *Scytinostroma*, *S. portentosum*. Although the genus is not monophyletic (Larsson 2007), *Scytinostroma* currently includes 25 names, one of which was added by Wang et al. (2020). The type of the genus and other species of *Scytinostroma* with globose basidiospores were placed in “group 1” by Boidin and Lanquetin (1987). To this group these authors also refer *Stereofomes nodulosus*, the lectotype of *Stereofomes* selected by Donk (1956), because it was the only species that included a description as later discussed and accepted by Boidin and Lanquetin (1987). They recognized the synonymy of *Scytinostroma* with *Stereofomes* and suggested that *Scytinostroma* should be conserved over *Stereofomes,* although this was never formally done. Among the 12 names placed in *Stereofomes*, only four remain in that genus. Of these four synonymous generic names, *Scytinostroma* is the most well-known (GSS *Scytinostroma* = 612, *Licrostroma* = 30, *Stereofomes* = 13, *Michenera* = *ca* 50, but confused with other meanings of the word). Despite its polyphyletic nature, it seems least disruptive to protect the generic name *Scytinostroma* for the type *S. portentosum* and its relatives.

A final note is that *Artocreas*, lectotypified by *Artocreas micheneri* by Massee (1888), was based on the same find as *Michenera artocreas*, and may have been an unintentional flipping of the two binomial parts (cf. Donk 1962) but are clearly valid names. Considerable confusion was sown by Berkeley throughout the publication history of *Artocreas* by one lapsus calami after another. Berkeley in Berkeley and Curtis (1869) first published the name *Michenera artocreas*. Then he published *Artocreas* with *Artocreas micheneri* and *A. poriniiforme* (Berkeley and Broome 1875). This was followed by an admission of error (Berkeley and Broome 1876) that introduced more errors, namely that *Artocreas* is the same as *Michenera*, but he spelled *Artocreas* in two different ways with two different author citations, namely “*Artocreas*, B. & Br.” and “*Artrocreas*, B. & C.”. Massee (1888) further clarified and then muddied the waters by first lectotypifying *Artocreas* by *A. micheneri*, then declaring after a long discussion on *Artocreas poroniiforme*, “Under the circumstances it has been considered advisable to propose the genus *Artocreas* as the type of a new order, occupying a position exactly intermediate between the *Nidulariaceae* and the *Hymenogastreae*”. He then proceeds to name a new order *Matulales* (as *Matuleæ*) with a new genus *Matula*, typified by *Matula poroniiforme*, and not by *Artocreas*.

***New combinations:***

***Scytinostroma artocreas*** (Berk. & M.A. Curtis) K.-H. Larss., **comb. nov.**

MycoBank MB 837870

*Basionym*: *Michenera artocreas* Berk. & M.A. Curtis, J. Linn. Soc., Bot. **10**: 333. 1868 “1869”.

*Synonyms*: *Corticium subgiganteum* Berk., Grevillea **2**(13): 3. 1873.

*Licrostroma subgiganteum* (Berk.) P.A. Lemke, Canad. J. Bot. **42**: 763. 1964.

*Artocreas micheneri* Berk. & M.A. Curtis, in Berkeley & Broome, J. Linn. Soc., Bot. **14**: 73. 1873 “1875”.

*Aleurodiscus orientalis* Lloyd, Mycol. Writ. **6**(62): 927. 1920.

*Aleurodiscus reflexus* Yasuda, Bot. Mag. (Tokyo) **35**(420): 269. 1921.

*Globuliciopsis lindbladii* Hjortstam & Ryvarden, Syn. Fungorum **22**: 19. 2007.

***Scytinostroma incrustatum*** (S.H. He et al.) K.H. Larss., **comb. nov.**

MycoBank MB 838392

*Basionym: Michenera incrustata* S.H. He et al., Nova Hedwigia **108**: 199. 2018 “2019”.

***Scytinostroma nodulosum*** (Rick) K.H. Larss., **comb. nov.**

MycoBank MB 837871

*Basionym*: *Stereofomes nodulosus* Rick, Egatea **13**: 435. 1928.

### Use *Sistotrema* Pers. 1794 (S) rather than *Ingoldiella* D.E. Shaw 1972 (A)

In sanctioning *Sistotrema*, Fries (1821) included only *S. confluens* Pers. 1794, although listing *Hydnum sublamellosum* Bull. 1787 as a synonym. Persoon (1794) had previously described *Sistotrema* including *S. confluens* with *Hydnum sublamellosum* as a synonym as well as *S. cinereum* and *Boletus unicolor. Sistotrema hamatum* was described as the sexual morph of *Ingoldiella hamatum*, the type of *Ingoldiella,* according to Nawawi and Webster (1982) who obtained both morphs in pure culture from partially submerged decaying leaves collected in a stream in Malaysia. Although *S. hamatum* has not been sequenced, it has been widely accepted in *Sistotrema*, as congeneric with *Sistotrema confluens*, thus *Sistotrema* and *Ingoldiella* are synonyms. The large genus *Sistotrema* is classified in *Hydnaceae*, *Cantharellales,* with close relationships to *Clavulina*, *Hydnum*, and *Membranomyces*. It is considered polyphyletic (Moncalvo et al. 2006; Bernicchia and Gorjón 2010) or paraphyletic (Larsson 2007). Zhou and Qin (2013) determined that a monophyletic *Sistotrema* can be divided into subclades that include the type plus 13 members of the genus as well as other genera, however, *S. hamatum* was not included in this study. *Sistotrema* is more commonly used than *Ingoldiella* (GSS *Sistotrema* = 1250, *Ingoldiella* = 91) and includes over 200 names while *Ingoldiella* includes only three names. Given the widespread use of *Sistotrema*, the greater number of names, and its priority, we recommend the use of *Sistotrema*.

### Use *Sterigmatosporidium* G. Kraep. & U. Schulze 1983 (A) rather than *Cuniculitrema* J.P. Samp. & R. Kirschner 2001 (S)

*Sterigmatosporidium polymorphus*, the type and only species in *Sterigmatosporidium*, was described for a species later determined to be the asexual morph of *Cuniculitrema polymorpha*, the type and only species of *Cuniculitrema* (Kirschner et al. 2001), thus these generic names are synonyms. *Sterigmatosporidium* was initially considered to be related to *Sterigmatomyces* (Kraepelin and Schulze 1982), the latter genus typified by *S. halophilus,* and now shown to belong in *Pucciniomycotina* (Aime et al. 2006, 2018). Liu et al. (2015) included *Sterigmatosporidium polymorphus* (as *Cuniculitrema polymorpha*) in their molecular study of *Tremellomycetes* and placed this species near *Fellomyces* in *Agaricomycotina*. *Sterigmatosporidium* is used more extensively than *Cuniculitrema* (GSS *Sterigmatosporidium* = 186, *Cuniculitrema* = 60). Given that both genera are monotypic but *Sterigmatosporidium* has priority and is more widely used, we recommend the use of *Sterigmatosporidium*.

### Use *Subulicystidium* Parmasto 1968 (S) rather than *Aegeritina* Jülich 1984 (A)

The monotypic *Aegeritina* is typified by *A. tortuosa*, now considered a synonym of *Subulicystidium longisporum,* the type of *Subulicystidium* (Kendrick and Watling 1979), thus these generic names are synonyms*.* Because it has priority, includes 11 names, and is widely used (GSS *Subulicystidium* = 248, *Aegeritina* = 19), we recommend the use of *Subulicystidium*.

### Use *Tomophagus* Murrill 1905 (S) rather than *Thermophymatospora* Udagawa, Awao & Abdullah 1986 (A)

The generic name *Tomophagus,* typified by *T. colossus,* was established for an unusual species often referred to as *Ganoderma colossus* (Fr.) C.F. Baker 1920*.* Hong and Jung (2004) were the first to show that *G. colossus* fell outside *Ganoderma*. Le et al. (2012) recognized this species in *Tomophagus* and described a second species of *Tomophagus*, *T. cattienensis*. The monotypic *Thermophymatospora,* typified by *T. fibuligera,* was described for an asexual basidiomycete, which was later determined to be the asexual morph of *Tomophagus colossus* as *G. colossus* (Adaskaveg and Gilbertson 1989), thus *Tomophagus* and *Thermophymatospora* are synonyms. Given its priority, the greater number of names, widespread use (GSS *Tomophagus* = 115, *Thermophymatospora* = 19), and recent study, we recommend the use of *Tomophagus*.

### Use *Trechispora* P. Karst. 1890 (S) rather than *Osteomorpha* Watling & W.B. Kendr. 1979 (A)

The type of *Trechispora, T. onusta,* is now regarded as a synonym of *T. hymenocystis* (Larsson 1994). When Watling and Kendrick (1979) validated the name *Osteomorpha,* typified by *O. fragilis*, they noted its association with *Trechispora farinacea*, a species name used at that time in a broad sense (Liberta 1973). Since then, a number of authors have suggested that *O. fragilis* is the asexual morph of *Trechispora farinacea* (Hjortstam et al. 1988; Mel’nik 2011). Later studies have shown that several *Trechispora* species are associated with an asexual morph similar to *Osteomorpha* (Larsson 1995; Miettinen and Larsson 2006), although it is most commonly seen with *T. stevensonii* (Berk. & Broome) K.H. Larss. 1995. Ordynets et al. (2015) showed that *T. farinacea* and *T. stevensonii* are distinct species and that both resolve within *Trechispora* together with the type of *Trechispora*, thus *Trechispora* and *Osteomorpha* are synonyms. *Trechispora* is a widely used genus with over 90 names (GSS *Trechispora* = 1490, *Osteomorpha* = 39) and has priority, while *Osteomorpha* includes only the type, thus we recommend the use of *Trechispora*.

### Use *Trimorphomyces* Bandoni & Oberw. 1983 (S) rather than *Anastomyces* W.P. Wu et al. & Gange 1997 (A)

In studying *Trimorphomyces* typified by *T. papilionaceus*, Kirschner and Chen (2008) concluded that the type of the monotypic genus *Anastomyces*, *A. microsporus*, was the asexual morph of *T. papilionaceus*, thus *Trimorphomyces* and *Anastomyces* are synonyms. Although both generic names include only one species name, *Trimorphomyces* is used more frequently (GS = 82) than *Anastomyces* (GS = 13). Given its priority and more common use, we recommend the use of *Trimorphomyces*.

### Protect *Tulasnella* J. Schröt. 1888 [June] (S) over *Hormomyces* Bonord. 1851 (A) and use rather than *Prototremella* Pat. 1888 [August] (S), *Hormisciopsis* Sumst. 1914 (A), or *Epulorhiza* R.T. Moore 1987 (A)

The generic name *Tulasnella* is typified by *T. lilacina*, regarded as a synonym of *T. violea* by Donk (1966), but not by Roberts (1994a, b), and includes 91 names. *Hormomyces* is typified by *H. aurantiacus* and includes seven names. The latter was often considered the asexual morph of *Tremella mesenterica* following the speculations of Saccardo (1916) and Bresadola (1932). This was never proven experimentally but was broadly accepted (e. g. McNabb 1969). After the cultural studies of *H. aurantiacus* by Tubaki (1976) and the description of the asexual morph of *T. mesenterica* by Pipolla and Kotiranta (2008), it was clear that this putative connection was incorrect. rDNA phylogenies by Mack et al. (2021) suggest that *H. aurantiacus* is a member of the same clade as *Tulasnella violea*, but is unlikely to be conspecific with it*. Hormisciopsis gelatinosa,* the only named species in that genus, is likely to be a synonym of *Hormomyces aurantiacus.* The generic name *Prototremella*, published in the same year as *Tulasnella*, is typified by *P. tulasnei*, now *Tulasnella tulasnei* (Pat.) Juel 1897. The synonymy of these two generic names and the selection of *Tulasnella* as the accepted name by Donk (1966) has never been questioned and is supported by Stafleu and Cowan (1976) and the monthly publication dates recorded in the *Journal de Botanique. Epulorhiza* with seven names is typified by *E. repens* based on *Rhizoctonia repens*. Warcup and Talbot (1967) identified the sexual morph of *R. repens* as *Tulasnella calospora*, with reference to Rogers (1933) and Olive (1957) both of whom used the name *T. calospora* for all *Tulasnella* collections with spores over 15 μm long, regardless of differences in spore shape. Roberts (1994a, b) re-examined type collections of *T. calospora* and *T. deliquescens* and distinguished the two species based on spore shape and size, noting that the illustration of “*Tulasnella calospora*” in Warcup and Talbot (1967) was misnamed and was actually *T. deliquescens*. The name *T. calospora* has continued to be used by many authors for the sexual morph of *E. repens*. Cruz et al. (2016) and papers cited therein could not resolve the synonymy of *T. calospora* and *T. deliquescens* and considered them to be distinct species as does Oberwinkler et al. (2017). Regardless, *T. calospora/T. deliquescens* and *T. violea* are congeneric (Cruz et al. 2016; Kristiansen et al. 2001; Moncalvo et al. 2006; Linde et al. 2017), thus *Tulasnella* and *Epulorhiza* are synonyms. Many of these species, especially *T. calospora/deliquescens,* are associated with the roots of orchids (Linde et al. 2017; McCormick et al. 2004; Weiss et al. 2004). Despite *Hormomyces* being an older name, it has rarely been used in the academic literature (GSS *Tulasnella* = 1629, *Hormomyces* = 42). Similarly, *Tulasnella* is more widely used than *Epulorhiza* (GSS = 875) and the two other obscure generic synonyms (GSS *Prototremella* = 35, *Hormisciopsis* = 9). Therefore, we recommend the use of *Tulasnella*.

*Epulorhiza* is already considered a taxonomic synonym of *Tulasnella* and several species, e. g. *E. amonilioides* and *E. anaticula,* were already transferred to that genus (Fujimori et al. 2019). The remainder are recombined in *Tulasnella* below:

***New combinations:***

***Tulasnella albertensis*** (Currah & Zelmer) J. Mack & P. Roberts, **comb. nov.**

MycoBank MB 32427

*Basionym*: *Epulorhiza albertensis* Currah & Zelmer, Rep. Tottori Mycol. Inst. **30**: 48. 1992.

***Tulasnella calendulina*** (Zelmer & Currah) J. Mack & P. Roberts, **comb. nov.**

MycoBank MB 832428

*Basionym*: *Epulorhiza calendulina* Zelmer & Currah, Canad. J. Bot. **73**: 1984. 1995.

***Tulasnella epiphytica*** (O.L. Pereira et al.) J. Mack & P. Roberts, **comb. nov**.

MycoBank MB 832427

*Basionym*: *Epulorhiza epiphytica* O.L. Pereira et al., Mycoscience **44**: 154. 2003.

***Tulasnella inquilina*** (Currah et al.) J. Mack & P. Roberts, **comb. nov.**

MycoBank MB 832430

Basionym: *Epulorhiza inquilina* Currah et al., Mycotaxon **61**: 338. 1997.

### Protect *Typhula* (Pers.) Fr. 1818 (S) over *Sclerotium* Tode 1790 (A)

The genus *Typhula,* lectotypified by *T. phacorrhiza* (Donk 1933), includes over 150 species. *Typhula* was originally published as *Clavaria* [unranked] *Typhula* by Persoon (1801) who included six species in the unranked infrageneric taxon. Fries (1818) listed four species when he recognized this taxon at the generic level. Donk (1954) noted that the proposed lectotype of *Typhula* by Clements and Shear (1931) of *T. sclerotioides* (Pers.) Fr. 1838 (syn. *Phacorhiza sclerotioides* Pers. 1822) was ineffective because this species was not included in the protologue or sanctioning work. The genus *Sclerotium,* typified by *S. complanatum,* was established for fungi producing asexual sclerotia. The typification of *Sclerotium* is explained by Donk (1962) in which the lectotypification by Clements and Shear (1931) followed by Cooke (1953) is accepted. The name *S. complanatum* is considered to be the asexual morph of *T. phacorrhiza* (Remsberg 1940), thus *Typhula* and *Sclerotium* are synonyms. Recently the diverse phylogenetic affinities of species of *Sclerotium* have been determined (Xu et al. 2010), many of which are members of *Ascomycota*. Given the confusion about the phylogeny of species of *Sclerotium* and the numerous species placed in the relatively well-defined genus *Typhula*, protection of the generic name *Typhula* is recommended.

### Protect *Typhulaceae* Jülich 1982 (S) over *Sclerotiaceae* Dumort. 1822 (A)

The type genus of *Sclerotiaceae* is *Sclerotium* Tode. *Sclerotiaceae* was first spelled ‘*Sclerotaceae*’ and correctly spelled by Link (1826). The name *Sclerotiaceae* has fallen out of use as a generalized family name for any sclerotial-based genera whereas the name *Typhulaceae* is useful for this phylogenetically defined group.

### Use *Waitea* Warcup & P.H.B. Talbot 1962 (S) rather than *Chrysorhiza* T.F. Andersen & Stalpers 1996 (A) and for some species previously placed in *Rhizoctonia*

The genus *Waitea*, typified by *W. circinata* (Warcup and Talbot 1962), mainly includes pathogens that cause diseases of monocotyledonous plants such as brown ring patch of turfgrasses, sclerotial rot and ear rot of corn, sheath spot of rice, and damping off and root rot of cereals and grasses, which typically occur in temperate regions (Gutierrez et al. 2007; Toda et al. 2007). The monotypic name *Chrysorhiza* was introduced for *Rhizoctonia zeae*, the asexual form of *Waitea circinata* (Stalpers and Andersen 1996), although this has been determined to be incorrect [see below]. *Chrysomyxa* has been little used (GSS *Waitea* = 1460, *Chrysorhiza* = 163), thus we recommend the use of *Waitea*.

The generic name *Rhizoctonia*, typified by *R. solani* (*Ceratobasidiaceae*, *Cantharellales*), is not congeneric with *W. circinata* (syn. *Rhizoctonia zeae)* (Vilgalys and Cubeta 1994), as *W. circinata* and several invalid names in *Rhizoctonia* belong in *Waitea* within *Corticiaceae* (De Priest et al. 2005; Ghobad-Nejhad et al. 2010). One new combination and three new species in *Waitea *are established below.

***New combination:***

***Waitea zeae*** (Voorhees) J.A. Crouch & Cubeta, **comb. nov.**

MycoBank MB 837872

*Basionym*: *Rhizoctonia zeae* Voorhees, Phytopathology **24**: 1299. 1938.

*Synonyms*: “*Chrysorhiza zeae*” (Voorhees) T.F. Andersen & Stalpers, Rhizoctonia-forming Fungi 58. 1996; nom. inval. (Art. 41.5).

*Moniliopsis zeae* (Voorhees) R.T. Moore, Mycotaxon **29**: 96. 1987.

*Rhizoctonia endophytica* var. *filicata* H.K. Saksena & Vaartaja, Canad. J. Bot. **38**: 938. 1960.

“*Waitea circinata* var. *zeae*” Toda et al., Plant Disease **89**: 536. 2007; nom. inval. (Arts. 39.1, 40.1).

Several studies have shown that *Rhizoctonia zeae* should be placed in *Waitea* as a species distinct from *W. circinata* (Chang and Lee 2016; de la Cerda et al. 2007; Gürkanli et al. 2016; Toda et al. 2005, 2007; Vojvodić et al. 2020); the new combination is therefore made here.

**New species:**

***Waitea agrostidis*** J.A. Crouch & Cubeta, **sp. nov.**

MycoBank MB 837874

*Synonym*: “*Waitea circinata* var. *agrostis*” S.J. Kammerer et al., Plant Disease **95**: 521. 2011; nom. inval. (Art. 39.1, 40.1).

*Diagnosis*: Light yellow colony on PDA differs from pinkish-white colonies of *W. circinata*, white-salmon pink colonies of *W. oryzae,* yellow-pink colonies of *W. prodiga,* and orange colonies of *W. zeae*. Irregularly shaped, dark brown sclerotia differ from pinkish or orange sclerotia of *W. circinata*, irregular, salmon-pink to orange sclerotia of *W. oryzae*, irregular to spherical salmon to yellow-salmon sclerotia of *W. prodiga,* and the subspheroid, orange sclerotia of *W. zeae*.

*Description*: Toda et al.*,* J. Gen. Plant Path*.* **73**: 385. 2007.

*Type*: Illustration in J. Gen. Plant Path. **73**: 383, fig. 1, 2007 (holotype) based on an unspecified collection made in Japan. Representative sequence: GenBank AB213567 (ITS) derived from a collection: Japan, Aichi, on *Agrostis stolonifera* L. var. *palustris*, June 1999, isolate NUK-3BG (deposition unknown).

An unidentified *Rhizoctonia* sp. was found causing a destructive new disease affecting *Agrostis stolonifera* and *Poa pratensis* turfgrasses in Japan; the disease is referred to as Waitea reddish-brown patch disease (Toda et al. 2007). Later authors referred to this *Rhizoctonia* sp. as “*Waitea circinata* var. *agrostis*” but the name was not validly published (Kammerer et al. 2011). Phylogenetic relationships based on DNA sequences show that this fungus is a member of the genus *Waitea* as a species distinct from *W. circinata* and other species in the genus (Kammerer et al. 2011; Toda et al. 2007), thus this new species is described here. Isolates utilized by Toda et al. (2007) could not be located, therefore an illustration is designated as the type and a reference sequence is selected from those obtained by Toda et al. (2007).

***Waitea oryzae*** J.A. Crouch & Cubeta, **sp. nov.**

MycoBank MB 837881

*Synonyms*: “*Rhizoctonia oryzae*” Ryker & Gooch, Phytopathology **28**: 238. 1938; nom. inval. (Art. 39.1).

“*Waitea circinata* var. *oryzae*” Toda et al., Plant Disease **89**: 536. 2007; nom. inval. (Arts 39.1, 40.1).

*Diagnosis*: White to salmon pink colony on PDA differs from pale yellow colonies of *W. agrostidis*, pinkish-white colony of *W. circinata*, yellowish pink colony of *W. prodiga,* and orange colony of *W. zeae*. Irregularly shaped, salmon-pink to orange sclerotia differ from irregular, dark brown sclerotia of *W. agrostidis*, pinkish or orange sclerotia of *W. circinata*, irregular to spherical, salmon to yellow-salmon sclerotia of *W. prodiga,* and subspheroid, orange sclerotia of *W. zeae*.

*Description*: Ryker & Gooch, Phytopathology **28**: 238. 1938; as “*Rhizoctonia oryzae*”.

*Type*: USA: *Louisiana*: Crowley: on stems of *Oryza sativa*, 22 Jul. 1938, *T.C. Ryker 3049* (BPI 455795 – holotype). Representative sequence: GenBank AB213589 (ITS) derived from a collection: Japan, Toyama, on *Oryza sativa*, isolate RoTTS (deposition unknown).

*Rhizoctonia oryzae* and *W. circinata var. oryzae* are invalid names used by plant pathologists to refer to the causal agent of leaf and sheath spots of grasses and cereals. Although neither of these two names were validly published, there is no doubt that this fungus is congeneric with *W. circinata* and distinct from other species in the genus (Chang and Lee 2016; de la Cerda et al. 2007; Gürkanli et al. 2016; Kammerer et al. 2011; Leiner and Carling 1994; Ryker and Gooch 1938; Sharon et al. 2006; Toda et al. 2005, 2007). Therefore, the new species *W. oryzae* is formally established here. The original collection made by Ryker and Gooch (1938) held by BPI has been chosen as the type specimen. In the absence of a sequence from the type, one of the five sequences included by Toda et al. (2007) under *R. circinata* var. *oryzae* is indicated as a reference sequence.

***Waitea prodiga*** J.A. Crouch & Cubeta, **sp. nov.**

MycoBank MB 837883

*Synonym*: “*Waitea circinata* var. *prodiga*” S.J. Kammerer et al., Plant Disease **95**: 521. 2011; as *‘prodigus’*; nom. inval. (Arts 39.1, 40.1).

*Diagnosis*: Yellow-pink colony on PDA differs from pale yellow colony of *W. agrostidis*, dark brown colony of *W. circinata*, white-salmon pink colony of *W. oryzae,* and orange colony of *W. zeae*. Irregular to spherical, salmon to yellowish salmon sclerotia differ from irregular, dark brown sclerotia of *W. agrostidis,* pinkish or orange sclerotia of *W. circinata*, irregular, salmon pink to orange sclerotia of *W. oryzae,* and subspheroid, orange sclerotia of *W. zeae*.

*Description*: Kammerer et al.*, Plant Disease***95**: 521. 2011.

*Type*: Illustration in Plant Disease **95**: 517, fig. 2a, 2b (second sclerotium from left), 2011 (holotype), based on the collection: USA: *Florida*: Fort Myers, on leaves of *Paspalum vaginatum* ‘Sea Dwarf’, 4 Jan. 2008, *S.J. Kammerer* 44 SK-PSA-TM4 (ITS GenBank HM597146).

*Waitea circinata* var. *prodiga* was proposed as the name of a novel fungal pathogen responsible for a basal rot disease first identified from *Paspalum vaginatum* (Kammerer et al. 2011). The name was not validly published, but the phylogenetic relationship of this organism with other species of *Waitea* and distinctive morphological characters support the separation of *W. prodiga* from other species in the genus. Therefore, *Waitea prodiga* is here formally established as a new species. Isolates used by Kammerer et al. (2011) were not lodged in a reference collection, therefore an illustration is designated as the type, and a reference sequence is selected from those obtained by Kammerer et al. (2011).

### Protect *Wolfiporia* Ryvarden & Gilb. 1984 (S) over *Gemmularia* Raf. 1819 (A), *Pachyma* Fr. 1822 (A), and *Tucahus* Raf. 1830 (A)

The generic name *Wolfiporia,* typified by *W. cocos,* was established for the fungus known as tuckahoe or *Poria cocos,* common throughout North America and regarded as the fungus used medicinally in Asia (Wang et al. 2013). A sclerotial morph of this fungus was described as *Sclerotium cocos* and placed in *Pachyma* as *P. cocos*, type of that genus, as typified and explained by Donk (1962). Although he chose the first species listed, he supports this decision citing that it was based on “a statement by Fries himself … under *Pachyma* …” . *Wolfiporia* was typified by *Poria cocos* F.A. Wolf, a now conserved name with a conserved type. Had it not been conserved, *Poria cocos* would, under Art. F. 8, be typified by the type of *Sclerotium cocos*. An earlier epithet for the sexual morph was discovered for this species, namely *W. extensa* (Ginns and Lowe 1983); however, prior to changes in the Code permitting one name for each fungus, Redhead and Ginns (2006) proposed to conserve the commonly used name *Poria cocos.* This was recommended for approval (Norvell 2008) and is now conserved (Wiersema et al. 2020). *Wolfiporia* itself is not conserved and is a later synonym of the sanctioned names *Gemmularia* and *Pachyma*. Wu et al. (2020) resurrected the generic name *Pachyma*, placing into synonymy the name *Wolfiporia*. They also distinguished between the commercially important eastern Asian cultivated fungus, formerly classified as *Poria cocos* or *Wolfiporia cocos,* and the North American fungi under those names. Wu et al. (2020) adopted the sanctioned name *Pachyma hoelen* Fr. for the commercial fungus in Asia. However, because they used a secondary source using an older Code, they overlooked the fact that the also sanctioned generic name *Gemmularia* predates *Pachyma*. They listed it as “*Gemmularia* Raf. per Steud.” dating from 1824 taken from Donk (1962) who was using a Code recognizing a starting date of 1821 for most fungi. In that same publication, Donk (1962) lectotypified *Gemmularia* with *G. rugosa* Raf. The latter species was said to be the tuckahoe of North America (Rafinesque 1819). Donk (1962) questioned whether *Gemmularia* was accepted taxonomically by Fries (1823, 1832), which would mean the name is not sanctioned, but this argument is not currently accepted. Additionally, Fries (1832), while having the name *Gemmularia* typeset in smaller letters, nonetheless gave a direct reference to the page of his 1823 volume, which he did not do for other generic names he did not recognize. We conclude that *Gemmularia* like *Pachyma* is sanctioned. Rafinesque (1830) published yet another generic name, *Tucahus*, citing *Gemmularia* and the two species that he included in *Gemmularia*, but only describing *T. rugosus*.

While seven names have been placed in *Wolfiporia,* Lindner and Banik (2008) demonstrated that *W. dilatohypha* is not congeneric with *W. cocos* with the former species placed basal to *Laetiporus*. Only five names have been introduced in *Pachyma*, of which three (*P. cocos*, *P. hoelen* and *P. pseudococos*) relate to currently accepted species, while *Pachyma tuber-regium* is the sclerotial state of *Pleurotus tuber-regium* and *Pachyma woermannii* was introduced for the sclerotium of *Lentinus woermannii*, which is considered by Pegler (1983) to be a synonym of *Pleurotus tuber-regium* (as *Lentinus tuber-regium*). *Wolfiporia* is more widely used than *Pachyma* (GSS *Wolfiporia* = 2040, *Pachyma* = 479, *Gemmularia* = 8, *Tucahus* = 3) and the other names remain obscure. More recently, the entire genome of *Wolfiporia cocos* was sequenced and all sequences deposited under this name (Lee et al. 2019). Given its greater use and greater number of names, we recommend *Wolfiporia* for protection.

**New combination**:

***Wolfiporia hoelen*** (Fr.) Y.-C. Dai & V. Papp, **comb. nov.**

*Basionym*: *Pachyma hoelen* Fr., Syst. Mycol. (Index): 125. 1832, nom. sanct.

MycoBank MB 838346

## Additional names to be protected:

### Protect Phanerochaete chrysosporium Burds. 1974 (S) over *Sporotrichum pruinosum* Gilman & E.V. Abbott 1927 (A)

Type: See Table 2.

*Phanerochaete chrysosporium* is widely known for its industrial use as a white rot fungus that breaks down the aromatic polymer lignin and thus is important in the degradation of wood products (Kersten and Cullen 2007; Matityahu et al. 2015). Recently, *Phanerochaete chrysosporium* was confirmed as a member of the genus *Phanerochaete s. str.* (Floudas and Hibbett 2015). The older names *Sporotrichum pruinosum* and *S. pulverulentum* have been regarded as the asexual morph of this species either as one or two species (Burdsall Jr and Eslyn 1974; Burdsall Jr 1985). James et al. (2011) concluded that *S. pulverulentum* was distinct from *S. pruinosum* with the later linked to and providing an earlier name for *P. chrysosporium*. Given its widespread use and importance to applied microbiology, the name *P. chrysosporium* is proposed for protection.

### Protect *Polyporus mylittae* Cooke & Massee 1892 (S) over *Mylitta australis* Berk. 1839 (A)

Type: See Table 2.

The genus *Mylitta* Fr. 1825 is of uncertain application, but has been used for various growths, at least some of which were fungal sclerotia. *Mylitta australis* was introduced by Berkeley (1839) for the large sclerotia produced by a polypore later described from sporophores as *Polyporus mylittae* (Cooke 1892). The connection was explicitly made in the protologue of the latter where it was stated that the sporophores of *P. mylittae* were ‘growing on *Mylitta australis’* (Cooke 1892). All subsequent authors including Cunningham (1965) have accepted *M. australis* as synonymous with *P. mylittae*. Núñez and Ryvarden (1995) expanded the circumscription of *Laccocephalum* McAlpine & Tepper, typified by *L. basilapidoides* McAlpine & Tepper, to include several other sclerotium-forming austral polypores, specifically *Laccocephalum mylittae* (Cooke & Massee) Núñez & Ryvarden based on *P. mylittae*. This species produces large sporophores after wildfires and has been frequently recorded from across Australia as both *Polyporus* and *Laccocephalum*, under the epithet *mylittae*, including as a target species for the Fungimap mapping scheme (Grey and Grey 2005). In *Polyporus*, the epithet *australis* is pre-occupied by *Polyporus australis* Fr. 1828, now *Ganoderma australe* (Fr.) Pat., but there is no such obstacle to the transfer of *M. australis* to *Laccocephalum*. However, the name *M. australis* has only ever been applied to the sclerotium, and little used since the discovery of the sporophores more than a century ago. Therefore, *P. mylittae* is proposed for protection over *M. australis* to allow the continued use of *L. mylittae*.

## Data Availability

No original data was obtained for this project.

## Footnotes

1 ** ICTF *Homobasidiomycetes* WG initial membership: Co-conveners Tom May & Scott Redhead, Members Nico Dam, Ursula Eberhardt, Karl-Henrik Larsson, Ludmila Marvanová, Luis Morgado, Bernard Moyersoen, Lorelei Norvell, Manon Neilen, Joost Stalpers, and Benjamin Stielow.
